# Distinct roles for MNK1 and MNK2 in social and cognitive behavior through kinase-specific regulation of the synaptic proteome and phosphoproteome

**DOI:** 10.1038/s41380-026-03483-w

**Published:** 2026-02-13

**Authors:** Rosalba Olga Proce, Maria Steinecker, Chiara Giacomelli, Erika Uddström, Anirban Chatterjee, Souhaila Wüsthoff, Luiz Gustavo Teixeira Alves, Oliver Popp, Tobias Pohl, Katie Maxwell, Lucie Hortmann, Severine Kunz, Philipp Mertins, Markus Landthaler, Daria Bunina, Hanna Hörnberg

**Affiliations:** 1 Max Delbrück Center for Molecular Medicine in the Helmholtz Association, Molecular and cellular basis of behavior, Berlin, Germany; 2 Freie Universität Berlin, Institute of Chemistry and Biochemistry, Berlin, Germany; 3 Max Delbrück Center for Molecular Medicine in the Helmholtz Association, Proteome Dynamics, Berlin, Germany; 4 Max Delbrück Center for Molecular Medicine in the Helmholtz Association, Systems biology of cardiovascular and neuronal pathologies, Berlin, Germany; 5 Humboldt University, Faculty of Life Sciences, Institute for Biology, Berlin, Germany; 6 Max Delbrück Center for Molecular Medicine in the Helmholtz Association, RNA Biology and Post-transcriptional Regulation, Berlin, Germany; 7 Max Delbrück Center for Molecular Medicine in the Helmholtz Association, Proteomics Platform, Berlin, Germany; 8 Max Delbrück Center for Molecular Medicine in the Helmholtz Association, Electron Microscopy Technology Platform, Berlin, Germany; 9 Berlin Institute of Health at Charité-Universitätsmedizin Berlin, Berlin, Germany; 10 Humboldt University, Institute for Biology, Berlin, Germany

**Keywords:** Neuroscience, Molecular biology

## Abstract

Local mRNA translation is required for adaptive changes in the synaptic proteome. The mitogen-activated protein kinase (MAPK) interacting protein kinases 1 and 2 (MNK1 and MNK2) have emerged as key regulators of neuronal translation, primarily through phosphorylation of the eukaryotic initiation factor 4E (eIF4E). The therapeutic benefits of targeting the MNKs are being investigated for nervous system conditions that affect translation, including autism and pain. However, it is still unclear if MNK1 and MNK2 regulate distinct aspects of neuronal translation and how the activity of each kinase is associated with the synaptic and behavioral features linked to MNK signaling. To examine the individual neurobiological functions of each kinase, we used knockout mice lacking either MNK1 or MNK2. We found that knockout of MNK1 and MNK2 leads to different social and cognitive behavioral profiles and distinct alterations of the cortical synaptic proteome, transcriptome, and phosphoproteome. Loss of MNK1 was associated with increased ribosomal protein expression, whereas deletion of MNK2 decreased the expression and phosphorylation of synaptic proteins. Together, our findings provide evidence for a high degree of functional specialization of the MNKs in synaptic compartments and suggest that pharmacological inhibition of individual MNKs may provide more specific targets for neurological disorders.

## Introduction

Precise regulation of mRNA translation allows cells to modulate protein expression levels globally and to restrict protein localization to a specific time or cellular compartment [1]. In neurons, spatiotemporal regulation of mRNA translation is necessary for learning and memory. Part of this translation occurs at the synapse, where local translation contributes to adaptive changes in the synaptic proteome [2–6]. As synapses are space-restricted and reside far away from the soma, recent evidence suggests that translation may be regulated differently at the synapse compared to the soma [1, 7].

Translation of most mRNAs is regulated at the step of initiation by the formation of a pre-initiation complex that involves recognition and binding of the m^7^GTP cap structure by the cap-binding eukaryotic initiation factor 4E (eIF4E) [8]. eIF4E activity is modulated by phosphorylation of a single residue, Ser209, by the serine/threonine kinases mitogen-activated protein kinase (MAPK) interacting protein kinases 1 and 2 (MNK 1 and 2) [9, 10]. These non-essential kinases are mainly activated by p38 and the extracellular signal-regulated kinase (ERK)/MAPK pathways, and eIF4E is one of the few MNK targets validated in vivo [9, 11]. Both MNK1 and MNK2 are expressed in the brain [12], and the MNKs have been implicated in a range of adaptive behaviors, including memory formation, social behavior, and depressive-like behavior [13–16]. Most of these functions have been attributed to MNKs’ phosphorylation of eIF4E. For example, MNKs regulate long-term potentiation (LTP) in the dentate gyrus via eIF4E phosphorylation-mediated release of cytoplasmic FMR1-interacting protein 1 (CYFIP1) from the cap [17–19]. Recently, MNKs were found to also regulate neuronal translation via phosphorylation of the brain-specific synaptic Ras GTPase-activating protein 1 (Syngap1), which acts upstream of mTOR-dependent translation [15]. MNK-Syngap1 signaling modulates hippocampal learning and memory independent of eIF4E phosphorylation, indicating that the MNKs can regulate synaptic plasticity via distinct mechanisms that lead to the translation of largely independent pools of mRNAs [15, 16].

Because of their status as the sole kinases phosphorylating eIF4E, there has been considerable interest in targeting the MNKs for the treatment of nervous system disorders that affect translation, including neurodevelopmental conditions and neuropathic pain [10, 13, 14, 20–22]. MNK inhibition or loss of function appears to be well tolerated: MNK1/2 double-knockout mice are viable, and pharmacological inhibition of MNKs typically does not elicit behavioral phenotypes in wild-type mice [11, 13–15, 22]. Most MNK inhibitors target both MNK1 and MNK2, but they vary in specificity and inhibitory activity against each MNK, which may account for their differences in efficacy and ability to rescue specific phenotypes [13–15, 22, 23]. To further develop the MNKs as drug targets, an unanswered question is to what extent there is any functional specialization of the different MNK proteins in the nervous system. Although structurally similar, MNK1 and MNK2 are known to differ in their activity and have distinct roles in some biological processes [24–27]. While both kinases phosphorylate eIF4E under basal conditions, studies suggest that MNK1 is the main kinase that regulates eIF4E phosphorylation in response to signaling, whereas MNK2 is constitutively active [11, 17]. In addition, MNK2, but not MNK1, can modulate translation via inhibition of eIF4G activation and crosstalk with the mTOR pathway [28–32]. Studies using single-knockout mice suggest that MNK1 may be the main isoform in the brain [17, 18]. However, transcriptomic data show that MNK1 and MNK2 are broadly expressed in the mouse and human brain [12, 33], and data from ribosome sequencing suggest that translation of MNK1 and MNK2 is enriched in the neuropil compared to the somata of neurons [34]. Whether there is any functional specialization of the MNK proteins in neurons and synapses has yet to be established.

Here, we set out to characterize the individual neurobiological functions of MNK1 and MNK2. We find that mice lacking MNK1 or MNK2 have distinct behavioral profiles with different social and cognitive phenotypes. Deletion of MNK1 and MNK2 has strikingly different effects on the synaptic proteome, phosphoproteome, and transcriptome, suggesting that each kinase has separate functions at the synapse. Overall, our findings suggest that individual targeting of the MNKs should be considered when developing therapeutic approaches for disorders affecting the nervous system.

## Materials and methods

### Mice

Wild-type, MNK1^KO^, MNK2^KO^, and MNK1/2^DKO^ mice of both sexes were used for this study. The MNK mice were obtained from RIKEN (RBRC01512, RBRC01513, RBRC01514), and all mice were kept on a C57BL/6j background. Animals were weaned at P21-P23 and group-housed (2-5 mice per cage) under a 12 h light–dark cycle with food and water ad libitum. All experiments were performed during the light cycle. The age and sex is reported in the method for each experiment. Sample size was estimated based on data from previous studies [14]. All experiments were carried out in accordance with European animal welfare law and were approved by the Berlin Landesamt für Gesundheit und Soziales (LAGeSo).

### Behavior

All animals were juveniles (postnatal day 26 – 33) at the start of the first behavior, and both sexes were used for all behavioral tests. A minimum of two independent cohorts from multiple litters was used for all genotypes. The experimenter was not blinded to the genotype, as the same individual conducted both animal handling and behavioral testing. The order of the tests was as follows: social habituation/recognition test, open field, novel object test, social olfaction test, and object habituation/recognition test, with 1-4 days between tests.

### Social or object habituation/ recognition task

This test was performed as previously described [14]. A fresh home cage without grid, food, and water was used for the experimental cage. The animals were acclimated to the cage for 30 min before the start of the test. For the first trial, a novel same-sex mouse (stimulus mouse: C57BL/6 juvenile mice, P21-P28) or an object (dice or toy car) was placed into the cage for 2 min, and the mice were left to freely interact. This was repeated for 4 consecutive trials with 5 min between trial intervals. On the 5th trial, a novel mouse (littermate to the stimulus mouse) or a novel object (dice or toy car) was introduced for 2 min. For the social stimulus, the interaction was scored when the experimental mouse initiated the action and when the nose of the animal was oriented toward the social stimulus mouse only. For the object stimulus, interaction was scored when the nose of the mouse was oriented 1 cm or less toward the object. The interaction time was used to calculate the recognition index as: (Interaction trial 5) - (Interaction trial 4). The total interaction time and recognition index were scored manually, whereas additional analysis of specific social behaviors and locomotion was performed computationally using DeepOF [35] (social habituation/recognition) and DLCAnalyzer [36] (object habituation/recognition). Two animals (one MNK1^KO^ and one MNK1/2^DKO^) were excluded from the social habituation/recognition task because of aggressive behavior.

### Social or object habituation/ recognition task scoring with DeepOF

Recordings of the social habituation/recognition task were acquired through GoPro Hero9 cameras. The videos were processed through DeepLabCut (v2.2.2). A multi-animal project was created in which 8 body parts per animal were labeled (nose, left ear, right ear, body center, left side, right side, tail base, tail end) according to the protocol in Nath et al. [37]. The tracked data from the mice were processed using DeepOF. We used the supervised annotation analysis as shown in Borders et al. [35], but we modified the parameter for close contact to 1 cm. The parameters listed were: Of note, the different behaviors are not mutually exclusive, so behaviors can co-occur as long as the criteria for both behaviors are fulfilled.

**Table:** DeepOF supervised behavioral parameters.

Behavior parameter	Description
Nose to nose	Distance between the nose of the two mice < 1 cm. This was measured for both mice.
Nose to tail	Distance between the nose of one mouse and the tale base of the other <1 cm. This was measured for both mice.
Nose to body	Distance between the nose of one mouse and any body part of the other mouse 1cm. This was measured for both mice.
Side by side	The interaction was automatically scored when two distance thresholds were fulfilled: the distance between the noses of the two mice were 4.5cm, and the distance between the tail base of the two mice were 4.5cm.
Side by side reverse	The interaction was automatically scored when the distance between the nose of one mouse and the tail base of the other was 4.5cm.
Sniffing	The behavior was scored when the nose of one mouse was moving fast (50 mm/s) toward the wall. Distance between nose and wall 1cm
Climbing	The behavior was scored when the nose of one mouse was reaching beyond the wall, with a distance 1cm.
Speed	Total distance travelled.

Description of the all the behavioral parameters used to analyze the social habituation/recognition task. The supervised annotations described in Borders et al. (2023) were used, but parameter for close contact was modified to 1 cm.

For the LDA analysis, the mean of trials 1-5 was used for all behavioral parameters, and only test-driven and dyadic behaviors were included.

### Open field

The animal was placed in a 50 cm x 50 cm open field arena (ActiMot system, TSE), and its movements were monitored for 10 min using the ActiMot automated tracking system. The arena was cleaned with 70% ethanol between trials. One animal (wild-type mouse) was excluded for technical reasons as the system failed to record the movement of the animal, and for two MNK1^KO^ animals, the system failed to track the last three minutes.

### Novel object recognition

The day after the open field test, the animals were placed back into the same arena (ActiMot system, TSE) containing two identical objects (glass flasks) for 5 min. After one hour, short-term memory was tested by exposing the animals to a familiar object (glass flask) and a novel object (Lego blocks of similar size to the glass flask) for 5 min. Investigation of the object was considered when the mouse’s nose was sniffing less than a centimeter from or touching the object. The discrimination ratio was calculated as follows: (Time spent investigating novel object - familiar object)/(total time investigating). Object interaction was scored manually, and locomotion was assessed using DLCAnalyzer.

### Locomotion analysis using DLCAnalyzer

DLCAnalyzer [36] was used to analyze the distance traveled during the object habituation/recognition task and the novel object recognition test using coordinates obtained from DeepLabCut. The source code is available at https://github.com/topohl/SLEAPanalyzer.

### Social olfaction test

The test animals were placed in a fresh home cage with a grid without food and water. A cotton swab was attached to the grid, and the animal was left to acclimate to the environment for 30 min. Odor habituation and recognition were tested using a cotton swab soaked with an odor. Each odor was presented three times for two minutes, with one minute in between trials. The odors were: water, banana, and almond, and social odors collected by dragging the cotton swab through a dirty cage from sex-matched wild-type mice. Water was used as a habituation and is not shown in the figure. Time spent sniffing the swab was manually scored, with the observers blinded to the genotype. Sniffing was scored when the nose was within 2 cm of the cotton swab.

### Z-score normalization

Z-scoring was used to normalize each behavioral test against the mean of the wild-type control. The MNK1/2^DKO^ mice were normalized separately from the MNK1^KO^ + MNK2^KO^ mice to allow for comparison between the two experimental groups in the linear discriminant analysis. The Z-score for each behavior was calculated as shown below, where X: every observation, µ: mean of the control group, and σ: standard deviation of the control group.

$$Z=\frac{X-{{{\rm{\mu }}}}}{\sigma }$$

### Fluorescent in situ hybridization

Fluorescent in situ hybridization was performed for *Mknk1, Mknk2, Slc17a7*, and *Gad1* by using RNAscope® Multiplex Fluorescent Reagent Kit v2 assay (Advanced Cell Diagnostics, 323136) with probes Mm-Mknk1- C3 mRNA (Advanced Cell Diagnostics, 1270101-C3), Mm-Mknk2-C2 mRNA (Advanced Cell Diagnostics, 1270111-C2), Mm-Gad1-Mus mRNA (Advanced Cell Diagnostics, 400951), Mm-Slc17a7-O2 (Advanced Cell Diagnostics, 501101), and Mm-Slc6a3 (Advanced Cell Diagnostics, 315441) Briefly, snap-frozen brains were cut into 16μm thick slices with a cryostat. The brain slices were put on glass coverslips (Thermo Fisher Scientific, 10149870, 25x75x1mm) and kept at -70 °C until further processing. The samples were rinsed with 1x PBS (2x) and, after that, fixed with 4% paraformaldehyde (PFA) in 1x PBS for 30 min at RT, followed by washing with 1x PBS (2x). Dehydration was performed using an ethanol gradient of 50%, 70%, and 2×100%. Hybridization of probes and subsequent signal amplification were carried out using hydrogen peroxide and protease III, followed by the reagents in the Multiplex Fluorescent Detection Reagents v2 kits (Advanced Cell Diagnostics (ACD); Hayward, CA) as described in the manufacturer’s protocol. The fluorophores used to detect the probes were: TSA Vivid Fluorophore kit 520 (Tocris Bioscience, 7523/1, 1:1500), TSA Vivid Fluorophore kit 570 (Tocris Bioscience, 7526/1, 1:1500) and TSA Vivid Fluorophore kit 650 (Tocris Bioscience, 7527/1,1:1500). The slices were stained with DAPI (Advanced Cell Diagnostics, 323136) and mounted using ProLong Glass Antifade Mountant (Thermo Fisher Scientific, P36982). For cortex and hippocampus, images were obtained on the IXplore Spin Confocal Imaging Microscope (model IX83; Olympus Corporation) using a 60x oil immersion objective (UPLXAPO60XO, numerical aperture 1.42, Evident Corporation). The images were analyzed using ImageJ by acquiring the mean intensity of *Mknk1* and *Mknk2* signals in *Gad1* or *Slc17a7* positive cells in the cortical and hippocampal areas. For the VTA, fluorescence images were acquired on a Keyence BZ-X800 microscope with BZ-X Viewer version 01.03.00.01 and using a PlanApo 60×/1.40 NA oil immersion objective with Immersol 518 F oil. Filter sets included DAPI, GFP, TRITC, and Cy5. The black balance area was set to “small” in unstained areas; white balance was off. Gain was set to +6 dB for all channels, haze reduction was off. Images were acquired at 12-bit depth, stitched using BZ-X800 Analyzer version 1.1.2.4, and exported as full-resolution TIFF files, and analyzed in ImageJ by counting the number of puncta in Slc6a3-positive cells. Three wild-type male mice 4-9 weeks old were used for cortex and hippocampus and three wild-type males 6 weeks old for VTA. Percentages were assessed for all three mice independently and then averaged for the pie chart.

### Bioorthogonal non-canonical amino acid tagging (BONCAT)

400 μm thick coronal slices were cut on a vibratome in ice-cold cutting solution (87 mM NaCl, 25 mM NaHCO_3,_ 2.5 mM KCl, 1.25 mM NaH_2_PO_4_, 75 mM sucrose, 0.5 mM CaCl_2_, 7 mM MgCl_2_, 10 mM glucose, equilibrated with 95% O_2_ / 5% CO_2_). Slices were immediately transferred to a storage chamber containing artificial cerebrospinal fluid (ACSF, 125 mM NaCl, 25 mM NaHCO_3_, 2.5 mM KCl), 1.25 mM NaH_2_PO_4_, 2 mM MgCl_2_, 2.5 mM CaCl_2_, 11 mM glucose, pH 7.4, constantly bubbled with 95% O2 and 5% CO2). Slices were maintained at 32 °C in ACSF for 45 min and then moved to an incubation chamber and incubated for an additional 3 h with 1mM L-Azidohomoalanine (AHA) (Jena Bioscience, CLK-AA005). At the end of incubation, slices were snap-frozen in liquid nitrogen and stored at −70 °C. Slices were lysed in lysis buffer (1xPBS, 0.5% SDS + protease inhibitor) 12x per mg with a dunce homogenizer and centrifuged for 15 min 14 000 g at 4 °C. BCA-Assay (Pierce Protein BCA-Assay Kit) was used to determine the protein concentration. 500 µg of protein was diluted in the lysis buffer to a final volume of 150 µl. The samples were sonicated for 30 sec with a Hielscher ultrasonicator, then treated with 20 mM Iodoacetamide for 1 h at RT in the dark. To perform the click-reaction, the following reagents were added to the samples and briefly vortexed after each addition: 127 µM TBTA, 3.75 mM copper sulfate (Jena Bioscience, CLK-MI004-50), 100 µm PEG4-biotine alkyne (Jena Bioscience, CLK-TA0105-25), 1 mM TCEP, and adjusted with 1xPBS + protease inhibitor to a final volume of 400 µl. The samples were incubated for 2 h at RT in the dark during constant rotation. After incubation, proteins were extracted using the methanol-chloroform method. The final pellet was resolubilized in 150 µl RIPA (150 mM NaCl, Tx100 1%, Sodium deoxycholate 0.5%, Tris pH8 50 mM, SDS 0.1%) and sonicated for 30 sec. AHA incorporation was measured by western blotting using an anti-Streptavidin IRDye 800CW antibody (LI-COR, 926-32230, 1:2000). The results were normalized to total protein concentration using MEMCODE Reversible Protein Stain Kit (Pierce, # PIER24580). 3- to- 20-week-old male and female mice were used. Data from all ages were pooled, as no age-dependent effects were observed for any genotype. The results were replicated in 4 (cortex) or 5 (synaptoneurosomes) blots.

### Synaptoneurosome isolation

Cortex was rapidly dissected and homogenized in oxygenated Krebs buffer (118.5 mM NaCl, 2.5 mM CaCl_2_, 1.18 mM KH_2_PO_4_, 1.18 mM MgSO_4_, 3.8 mM MgCl_2_, 24.9 mM NaHCO_3_, 212.7 mM Glucose in diethyl pyrocarbonate (DEPC) treated H_2_O) with protease and phosphatase inhibitors in a dunce grinder. For polysome profiling experiments, the Krebs buffer was supplemented with 100 µg/mL cycloheximide. For BONCAT experiments, synaptoneurosomes were isolated from snap-frozen brain slices. An aliquot of the homogenate was taken from the whole lysate and snap-frozen in liquid nitrogen. The rest of the homogenate was passed through 2×100 µm pre-wet nylon filter (Merck Millipore, NY1H02500) using an 18 G needle, followed by a second filtration with a 5 µm pre-wet filter (Merck Millipore, NY0502500). The lysate was centrifuged for 10 min 1000 g 4 °C. The synaptoneurosome pellet was washed 1x in 500 µl Krebs buffer. The resulting pellet was snap-frozen in liquid nitrogen and stored at -70 °C until further use.

### Polysome profiling

A 10-50% w/v sucrose gradient was prepared using the Biocomp Gradient Master with the following buffer: 20 mM Tris pH 7.5, 150 mM NaCl, 5 mM MgCl_2_, 100 µg/mL cycloheximide, and 1 mM DTT. Cortex and synaptoneurosomes pellets were homogenized in polysome lysis buffer containing 20 mM Tris pH 7.5, 150 mM NaCl, 5 mM MgCl_2_, 24U/ml TurboTM DNase (Ambion, #AM2238) 100 µg/ml cycloheximide, 1 mM DTT, 1%Triton X-100, 1x protease inhibitor (Pierce, #A32959), 40U/ml RNase inhibitor (Promega, #N2511). Cortical homogenate was centrifuged 1x and synaptoneurosomes 2x at 16’000 x g for 10 min at 4 °C, and the supernatant was loaded onto the sucrose gradient. An equal amount of RNA was loaded onto the gradients. The gradients were centrifuged for 1 h at 4 °C, 37’000 rpm in a SW55 Ti rotor. Polysome profiling was performed using the Biocomp Piston Gradient Fractionator equipped with Biocomp TRIAX™ Flowcell for UV 260 nm and Gilson Fraction Collector FC-203B with the following settings: PGF speed = 0.2 mm/s, 23 s/fraction for collection of ~20 fractions. RNA was isolated from all fractions using the Direct-zol RNA MicroPrep kit (Zymo Research, #R2062) with Trizol LS (ABP Biosciences, #FP313), and the presence or absence of 18S and 28S rRNA was used to confirm the start of the monosome peak. The area under the curve (AUC) of the monosome and polysome peaks was quantified using the R/shiny app IPPA with the experimenter blinded to the genotype, available on https://github.com/ChiaraGiacomelli/IPPA. The samples were from 4 independent experiments. 4- to 7-week-old male mice were used for all genotypes.

### Quantitative PCR

RNA was isolated from the cortical and synaptoneurosome input samples from the polysome profiling, as well as additional 4- to 8-week-old male mice treated in the same way, using the Direct-zol RNA MicroPrep kit (Zymo Research, #R2062) with Trizol LS (ABP Biosciences, #FP313) and reverse transcribed into cDNA using random primers and the SuperScript™ III Reverse Transcriptase kit (Life Technologies GmbH, #18080044). The cDNA was used to prepare triplicate or duplicate reactions for qPCR using the TaqMan™ Fast Advanced Master Mix (Applied Biosystems™, #4444557) with the following TaqMan primers: Rn18s Mm04277571_s1, GAPDH Mm99999915_g1. The qPCR was run on a CFX384 Touch Real-time PCR Detection system (Bio-Rad) with the following conditions: UNG incubation at 50 °C for 2 min, Polymerase activation at 95 °C for 20 s, denaturation step for 1 s at 95 °C; annealing/extension step for 20 s at 60 °C, for 40 cycles. Each sample was internally normalized to GAPDH, and fold change was quantified using the comparative CT method, comparing all knockout genotypes to the mean of wild-type control. The results were replicated in two independent runs.

### Western Blot and AlphaLISA immunoassay

Brain tissue from adult male and female mice (10–21-week-old) was homogenized in RIPA buffer (150 mM NaCl, Tx100 1%, Sodium deoxycholate 0.5%, 50 mM Tris pH8, SDS 0.1%), and complete protease and phosphatase inhibitors. The synaptoneurosome pellet was either lysed in AlphaLISA lysis buffer (1x PBS, 5 mM EDTA, Tx100 1%, protease and phosphatase inhibitors), or RIPA buffer with protease and phosphatase inhibitors. For cortex homogenized in Krebs buffer for synaptoneurosome isolation, the whole homogenate was further diluted in RIPA buffer with protease and phosphatase inhibitors. For all western blot samples, protein concentration was measured with BCA-Assay (Pierce Protein BCA-Assay Kit), and diluted to an equal concentration in RIPA buffer and 4xLDS sample buffer (mpage, MPSB-10ml). Immunoblotting was done with HRP-conjugated secondary antibodies and WesternBright Chemilumineszenz Substrat (Biozym #541020, 541004). The following primary antibodies were used: p-4EBP1 (Cell Signaling, #2855S, 1:1000), 4EBP1 (Cell Signaling, #9644S, 1:1000). p-eIF4E (Abcam, ab76256 1:500), eIF4E (Cell Signaling, #9742S, 1:2000), p-ERK1/2 (Cell Signaling, #4370, 1:500), ERK1/2 (Cell Signaling, #4695, 1:500), MNK1 (Cell Signaling, #2195, 1:500), p-S6 (Cell Signaling, #5364 1:1000), S6 (Cell Signaling, #2317 1:1000). Secondary antibodies were anti-Rabbit IgG (Cell Signaling, #7074 1:2000) and anti-mouse IgG (Cell Signaling, #7076 1:2000). Membranes were stained with MEMCODE Reversible Protein Stain Kit (Pierce, # PIER24580) to visualize total protein concentration. Signals were acquired using an image analyzer (Bio-Rad, ChemiDoc MP Imaging System), and images were analyzed using ImageJ and Bio-Rad Image Lab software. The total protein concentration was used as a loading control for all experiments. The results were replicated in a minimum of two blots per antibody.

eIF4E phosphorylation was measured using the AlphaLISA SureFire Ultra p-eIF4E (Ser209) Assay Kits (PerkinElmer) according to the manufacturer’s protocol. AlphaLISA signals were measured using a Tecan SPARK plate reader in the recommended settings.

### Immunohistochemistry and imaging

Animals (3-6 weeks old males and females, 3 per genotype) were transcardially perfused with 4% PFA in 1xPBS. Brains were post-fixed overnight at 4 °C, incubated in 30% sucrose in 1xPBS for 3 days, and frozen with 2-methylbutan on dry ice. 30 µm sections were cut with a cryostat, and floating sections were stored at -20 °C in 30% ethylene glycol and 30% glycerol in 1x PBS until use. Floating sections were washed 3×10 min with 1xPBS followed by blocking with 10% donkey serum in 1xPBS containing 0.05% Triton X-100 for one hour at room temperature. Primary antibodies were incubated overnight in 1%BSA in 1xPBS. The following day, sections were washed 3×10 min in 1xPBS and incubated with the secondary antibodies and DAPI (Sigma-Aldrich, #MBD0015-1ML, 1:10000) in 1%BSA in 1xPBS for two hours at room temperature. Sections were washed 3×10 min in 1xPBS and mounted onto microscope slides using ProLong Glass Antifade mountant (Life Technologies GmbH, #P36980). Images were obtained on the IXplore Spin Confocal Imaging Microscope (Olympus) using a 30x silicon immersion objective (UPLSAPO30XS, numerical aperture 1.05, Evident Corporation). Per brain slice, five to seven images were acquired, covering isocortex and piriform cortex. The images were first analyzed using QuPath (version 0.5.0, University of Edinburgh, UK) to detect ROIs using “Cell Detection” with the following parameters: requested pixel size, 0.5 μm; background radius, 8 μm; opening by reconstruction enabled; median filter radius, 0 μm; sigma, 1.5 μm; minimum area, 10 μm²; maximum area, 400 μm²; split by shape enabled; cell expansion, 2 μm; smooth boundaries enabled; and make measurements enabled, and then in ImageJ (version 1.54 f; National Institutes of Health) to measure fluorescent intensity. The mean per image was used for statistical analysis. The following antibodies were used: mouse-anti riboRNA [Y10b] (Abcam, #ab171119, 1:50), guinea pig anti-NeuN (SYSY, #266004, 1:1000), Alexa Fluor® 488 AffiniPure Donkey Anti-Mouse IgG (H + L) (Jackson, #715-545-150, 1:1000), and Alexa Fluor 647-conjugated AffiniPure Donkey Anti-Guinea Pig IgG (H + L) (Jackson, #706-605-148, 1:1000).

### Electron microscopy

Mouse brain tissue coronal 600 µM slices were cut on a vibrotome and immediately fixed by immersion in 4% (w/v) paraformaldehyde plus 2.5% (v/v) glutaraldehyde in 0.1 M phosphate buffer for 3 h at room temperature (RT) followed by 2 days at 4 °C. Samples were processed using a modified version of the rOTO protocol by Deerinck et al., 2022 [38]. Sections were post-fixed with reduced 1% (v/v) osmium tetroxide (2% (w/v) aqueous sodium tetroxide + 1.5% (w/v) potassium ferrocyanide in 0.1 M phosphate buffer) for 60 min on ice, washed with 0.1% (w/v) aqueous thiocarbohydrazide, followed by incubation in 2% (v/v) osmium tetroxide for 90 min at RT. Final contrast was achieved by an incubation in 2% (w/v) uranyl acetate overnight at 4 °C. After dehydration through a graded series of acetone, embedding was done in durcupan resin. Ultrathin resin sections (80 nm) were collected on wafers and stained with 3% lead citrate. Imaging was done at 2 kV, 0.2 nA using BSE with the DBS detector and the Helios Hydra 5CX (Thermo Fisher / FEI, The Netherlands). Acquisition was made with the MAPS software package, version 3.27 (Thermo Fisher / FEI, The Netherlands). Somatosensory cortex was chosen as a representative cortical region. For quantification, 4×4 tile sets of 10 different positions were used, which covered an area of 4.1×15.3 µm with a pixel size of 674 pm / px. PSD length and thickness were measured manually using ImageJ v1.53t with the experimenter blinded to the genotype. Synapses were defined by postsynaptic densities in close proximity to presynaptic boutons containing synaptic vesicles. 5-week-old male mice were used, two wild-type, three MNK1^KO^, and three MNK2^KO^ mice.

### TMT mass spectrometry

Global proteomes and phosphoproteomes of cortical homogenate and synaptoneurosomes were analyzed using TMT (Thermo Fisher Scientific) isobaric labels combined with deep fractionation, as described in Mertins et al., 2018 [39]. Briefly, synaptoneurosome pellets and whole homogenate were lysed in SDS buffer (25 mM HEPES, 2% SDS, protease, and phosphatase inhibitors) and boiled at 95 °C for 3 min. Peptides were cleaned up and digested with trypsin using the SP3 protocol as previously described [40]. An amount of 100 µg of each peptide sample was subjected to TMTpro 18-plex (Thermo Fisher Scientific) labeling with randomized channel assignments. Quantitation across two TMT plexes was achieved by including an internal reference derived from a mixture of all samples. Samples were fractionated using an UltiMate 3000 Systems (Thermo Fisher Scientific) into 24 fractions for proteome analysis and 12 fractions for phosphoproteome analysis. For phosphoproteome analysis, the peptides were subjected to phosphopeptide enrichment using an AssayMAP Bravo Protein Sample Prep Platform (Agilent Technologies). All samples were measured on an Exploris 480 orbitrap mass spectrometer (Thermo Fisher Scientific) connected to an EASY-nLC system 1200 system (Thermo Fisher Scientific). 4-week-old male and female mice (two from each sex per genotype) were used for all TMT experiments.

For analysis, MaxQuant version 2.1.4.0 [41] was used, employing MS2-based reporter ion quantitation. Carbamidomethylation was set as a fixed modification and acetylated N-termini as well as oxidized methionine as variable modifications. For phosphoproteomics analysis, phosphorylation on serine, threonine and tyrosine was enabled as a variable modification. A PIF filter was applied with a threshold value of 0.5. For database search, a Uniprot mouse database (2022-03) plus common contaminants were used. Proteins with less than 2 peptides were excluded from the analysis. Corrected log2-transformed reporter ion intensities were normalized to the internal reference samples and further normalized using median-MAD normalization before applying two-sample moderated t-tests (limma) [42]. P-values were adjusted using the Benjamini-Hochberg procedure. The data was further analyzed using Protigy 1.1.5 (https://github.com/broadinstitute/protigy/), using two-sample mod t-test without group-wise normalization. An adjusted p-value < 0.05 was used for significance. Visualization of proteomic data was performed using Protigy v1.1.5 (PCA, heatmap of protein expression), GraphPad Prism 10.2.1 and in R studio (2023.12.1, R version 4.4.4) using the package corrplot (logFC correlation). For visualization of specific phosphosite intensities with multiplicities, a mean was used.

### Protein gene ontology and gene set enrichment analysis

Gene set enrichment analysis (GSEA) was performed using GSEA v4.3.2 [43] with 10 000 permutations. GO terms were collected from the mouse MSigDB database for canonical pathways or cellular components. LogFC of all proteins were used as gene ranks. Significance was set as FDR < 0.25, and only GO terms significant in MNK1^KO^ or MNK2^KO^ were used for further analysis. Hierarchical clustering was done by calculating the euclidean distance between the NES in R studio using the packages pheatmap and dichromt, with the method set as ward.d2. The number of clusters was determined using Nbclust. ggplot2 was used for GSEA visualization, and the heatmaps showing logFC of the individual GO terms were done using Morpheus [44]. To compare the distribution of ribosomal proteins, a random set of the same number of proteins as ribosomal proteins was generated from the total protein expression. PTM-SEA was performed using ssGSEA2.0 in R studio, with the mouse PTMsigDB signature set [45], with logFC as the rank input list. The number of permutations was set to 10 000, and significance was set to FDR < 0.05. Gene ontology was performed on phosphosites with a logFC value above 1.2 or below -1.2 using Enrichr [46], with all proteins with detected phosphosites set as background. Significance was set to p.ajust<0.05. P-value was adjusted using the Benjamini-Hochberg method. SynGO analyses were performed using the SynGO web page (version 1.2) with default settings [47]. ggplot2 was used for visualization. Venn diagrams were created using BioVenn [48].

### RNA-Sequencing and enrichment analysis

Samples for mRNA-seq experiment were collected from 3 four-week-old male mice for each knockout and wild-type genotypes, used as biological replicates in the subsequent analyses. Frozen synaptoneurosomes were resuspended in 600 mL TRIzol using a 22 G syringe and were centrifuged for 10 min 13 000 g 4 °C. Total RNA was isolated using the Direct-zol RNA microprep kit (Zymo Research, #R2062) and treated with DNAse, following the manufacturer’s instructions. Total RNA samples were quantified using a Qubit Fluorometer, and RNA integrity was checked on a TapeStation (Agilent). Double-indexed stranded mRNA-Seq libraries were prepared using the ILMN Stranded mRNA Library Prep Kit (Illumina, #20040534), starting from 250 ng of input material according to the manufacturer’s instructions. Libraries were equimolarly pooled based on Qubit concentration measurements and TapeStation size distributions. The loading concentration of the pool was determined using a qPCR assay (Roche, #7960573001). Libraries were then sequenced on the Illumina NovaSeq X Plus platform using PE100 sequencing mode, with a target of 50 million reads per library. RNA-seq reads were adapter trimmed using Trimmomatic v.0.39 and aligned to the mouse genome (mm10) with STAR aligner version 2.7.8a (https://github.com/alexdobin/STAR) using default parameters. Gene counts were produced with a featureCounts function from the subread v.2.0.3 and Ensembl GRCm39.109 annotation of all mouse genes. Differential expression analysis was performed using an R package DESeq2 v.1.44.0 for each mutant independently. Significantly differential genes were identified with an adjusted p-value threshold of 0.05. Gene ontology and gene set enrichment analyses were performed using R package clusterProfiler v.4.10.1 and canonical pathway database m2.cp.v2023.1.Mm.symbols for mouse genes, with significance set to adjusted p-value of <0.05. For GSEA comparison with the protein dataset, significance was set to the same adjusted p-value of <0.25, and only pathways present in both datasets were compared. Volcano plots were generated using EnhancedVolcano v.1.20.0 R package. K-means clustering was performed using stats base package in R v.4.3.2 with 20 clusters since the predicted number of clusters (3) did not achieve satisfactory group splitting by visual assessment. To order clusters according to their expression patterns, the mean expression profile was calculated across all conditions for each of the 20 k-means clusters. These cluster-level average profiles were then compared using pairwise Pearson correlations, and hierarchical clustering was performed on the resulting distance matrix. The dendrogram from this analysis was used to reorder the clusters on the heatmap visualization. Gene ontology was performed on the resulting cluster groups using the R package clusterProfiler v.4.10.1, with significance set to adjusted p-value of <0.05.

### Statistical analysis

Statistical analyses were performed with the GraphPad Prism software (version 10.2.1) or in R studio. All animals were used for further analysis unless otherwise stated. The sample size (n) for each experiment is listed in the figures and figure legends. When comparing a single factor, normality was tested using the Shapiro-Wilk test. When normally distributed, the data were analyzed using an unpaired two-tailed t-test, one-way ANOVA or repeated measures (RM) ANOVA, followed by Tukey’s post-hoc test. Homogeneity or equality of variance was confirmed using an F-test (t-test) or the Brown-Forsythe test (ANOVA). For non-parametric tests, two-tailed Mann-Whitney test or Kruskal-Wallis test followed by Dunn’s multiple comparison test were used. For comparison of multiple factors, two-way or three-way ANOVA or RM ANOVA with the Geisser-Greenhouse correction was used, followed by Tukey’s or Šidák’s multiple comparison test. Grouped data with missing values were analyzed using a mixed-effect model with the Geisser-Greenhouse correction followed by Tukey’s multiple comparison test. 3-way ANOVA was calculated using the R package ezANOVA(ez). Correlation was assessed using Pearson’s correlation. Outliers were identified using Rout Q = 0.1%, which led to the removal of one MNK1/2^DKO^ sample from Supplementary Figure 7G. Differences in frequency distribution were assessed using the Kolmogorov-Smirnov test. Linear discriminant analysis (LDA) was done using the package Mass in the R environment. Missing values (open field from 1 wild-type and last three minutes for 2 MNK1^KO^ mice) were imputed using the median from the same genotype. The last trial was missing from one MNK1/2^DKO^ mouse for the locomotion analysis of the object habituation/dishabituation test; here, the mean of the first four trials was used as input. Data are represented as the mean ± s.e.m., with the exception of violin plots where the median and quartiles are shown. Significance was set at P < 0.05. Detailed statistical information for all figures is shown in Supplementary Table 7.

## Results

### Deletion of MNK1 or MNK2 causes specific behavioral phenotypes

**Fig. 1:**
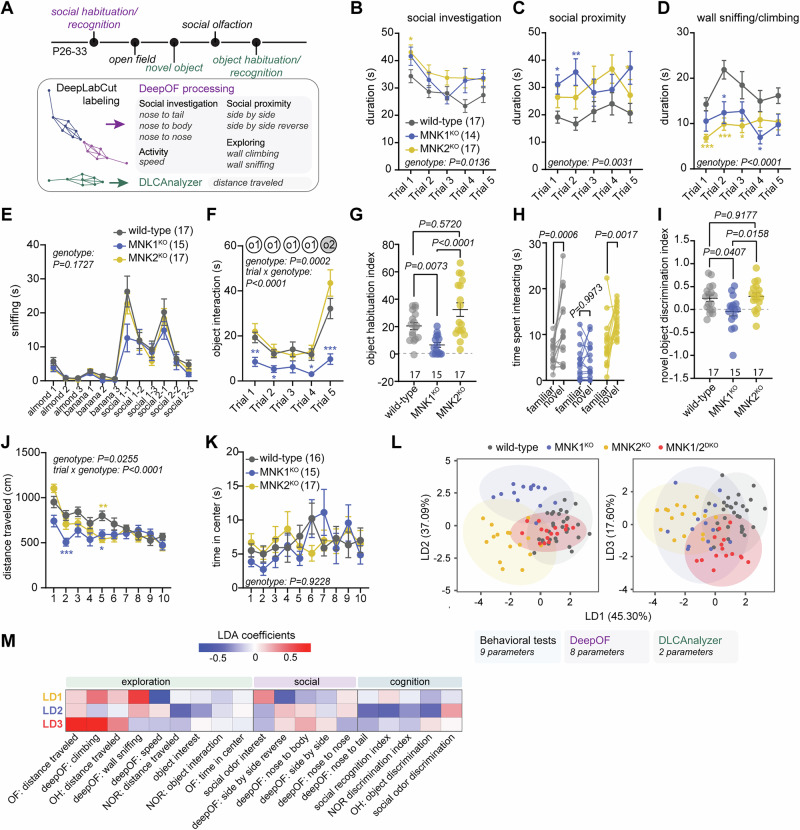
Mice lacking MNK1 or MNK2 have distinct behavioral profiles. (**A**) Order of behavioral tests and the corresponding analysis methods. (**B-D**) Time spent in social interaction (sum of nose to nose, nose to tail, nose to body, **B**), social proximity (sum of side by side by side and side by side reverse, **C**), and wall climbing/sniffing (sum of wall climbing and wall sniffing, **D**) in the five-trial social habituation/recognition test as quantified by DeepOF. (**E**) Time spent sniffing each odor in the social olfaction habituation test. (**F-G**) Mean object interaction time (**F**) and recognition index (**G**) in the object habituation/recognition test. o1=first object stimulus, o2=second object stimulus. (**H-I**) Time spent interacting with the familiar and novel object (**H**) and novel object discrimination index (**I**) in the novel object recognition test. (**J-K**) Distance traveled (**J**) and time spent in center (**K**) per minute in the open field test. (**L**) LDA of 19 behavioral parameters and (**M**) the LDA coefficients for LD1, LD2, and LD3. OF=open field, NOR=novel object recognition, OH=object habituation/recognition. Error bars show s.e.m. P values: *<0.5, **<0.01, ***<0.001 relative to wild-type. Significance was determined by mixed-effect model followed by Tukey’s post-hoc test for B, C, D, J, K, two-way RM ANOVA followed by Tukey’s post-hoc test for E, F, and Šidák’s post-hoc test for H, Kruskal-Wallis test followed by Dunn’s multiple comparison test for G, and one-way ANOVA followed by Tukey’s post-hoc test for I.

To examine the individual functions of the MNKs, we took advantage of knockout mice lacking either MNK1 or MNK2 and examined their behavioral phenotype. We performed several behavioral tests covering social, cognitive, and exploratory behaviors (Fig. 1A). All behaviors were videotaped to allow for automated annotation and motion tracking. Social interaction, habituation, and novelty preference were examined using the five-trial social habituation/recognition test (Supplementary Figure 1A). In this test, an unfamiliar sex- and age-matched conspecific is presented to the test mouse for four trials to examine social interest and habituation. On the fifth trial, a novel mouse is introduced to examine social recognition. We found that mice lacking MNK1, but not MNK2, exhibited a reduced interest in the novel mouse on the fifth trial compared to wild-type mice (Supplementary Figure 1B-C). While examining the videos from the social habituation/recognition test, we noticed differences in the types of social interactions observed in the MNK1 and MNK2 knockout mice. To quantify this further, we used DeepOF, an open-source system for deep social phenotyping of two freely interacting mice [35]. We first trained DeepLabCut [49] to recognize both animals during the two-minute-long videos and then analyzed all trials using the DeepOF supervised pipeline for automatic annotation of dyadic and individual behaviors (Fig. 1A). We found a slight but significantly higher overall social interaction in the MNK2^KO^ mice, primarily driven by increased nose-to-body contact (Fig. 1B, Supplementary Figure 1D). Both MNK1^KO^ and MNK2^KO^ mice spent significantly more time in close proximity to the stimulus mice without directly interacting (Fig. 1C, Supplementary Figure 1E), and significantly less time exploring the edges of the cage (Fig. 1D, Supplementary Figure 1F). There was no difference in the overall distance travelled (Supplementary Figure 1F) or in the social behavior of the stimulus animal (Supplementary Figure 1G-H). MNK1^KO^ mice also showed a tendency to reduced interest in social odors, but not nonsocial odors, in a social odor recognition task (Fig. 1E).

We next assessed object interaction and memory in two separate tests: the five-trial object habituation/recognition task and the novel object task. MNK1^KO^ mice had significantly reduced interaction with the first object in the habituation/recognition task compared to wild-type mice, and significantly reduced interest in the novel object presented on the 5^th^ trial (Fig. 1F-G). The reduced object recognition in MNK1^KO^ mice was also seen in the novel object task (Fig. 1H-I). Interestingly, mice lacking MNK2 showed increased interest in the novel object compared to wild-type mice in the object habituation/recognition task (Fig. 1F-G). In the open field test, both MNK1 and MNK2 knockout mice showed a slight decrease in distance traveled compared to wild-type mice, which was most pronounced during the first minutes of the test, but no change in time spent in the center (Fig. 1J-K). To examine if reduced locomotion could contribute to the observed phenotypes in the object recognition tests, we tracked the movement of the mice using DeepLabCut and analyzed their speed and distance traveled using DLCAnalyzer [36]. MNK1^KO^ mice showed reduced distance traveled during the novel object recognition test, but not during the object habituation/recognition test (Supplementary Figure 1I-J). There was no change in the locomotion of MNK2^KO^ mice for either test. Taken together, this suggests that MNK1^KO^ mice display reduced locomotion in a novel environment, but not in a home-like cage, and argues against reduced mobility as a contributing factor to the cognitive phenotypes. Mice from both sexes were used for all tests, and we observed no main effect of sex in any test (Supplementary Figure 2, Supplementary Table 1 for detailed analysis of sex and genotype interactions).

We next examined the behavioral phenotypes of mice lacking both MNK1 and MNK2 (MNK1/2^DKO^ mice) to assess possible redundancies of each kinase. Previous studies have shown deficits in spatial memory, long-term memory, and activity in MNK1/2^DKO^ mice, but no change in social behavior [15, 16, 50]. We found no differences in the social or short-term memory tests compared to wild-type mice (Supplementary Figure 3A-G, J-N), but a significant reduction in distance traveled in the open field and the object habituation/recognition test (Supplementary Figure 3H, O-P). There was no difference in time spent in the center in the open field test (Supplementary Figure 3I). To better understand the functional relationship between each genotype, we performed a linear discriminant analysis (LDA) using a total of 19 behavioral measures: 9 from classical assays, 8 test-mouse parameters from DeepOF, and 2 from DLCAnalyzer (Fig. 1L, all parameters listed in Supplementary Table 2). The first linear discriminant (LD1), which accounted for 45.30% of the total variance, primarily separated MNK2^KO^ mice from the other genotypes and was predominantly related to exploration during the social habituation test and passive social interaction (Fig. 1L-M). The second axis (LD2, accounting for 37.09% of the total variance) was strongly associated with performance in the cognitive tests and separated MNK1^KO^ mice from all other genotypes (Fig. 1L-M). MNK1/2^DKO^ mice overlapped with wild-type mice on LD1 and LD2 but showed distinct characteristics for LD3, which was mainly related to the distance travelled in the open field. Together, these results suggest that MNK1 and MNK2 have distinct contributions to social and cognitive behaviors, but have overlapping functions in regulating activity, which results in exaggerated hypoactivity in the MNK1/2^DKO^ mice.

### MNK1 and MNK2 are expressed in overlapping neuronal cell types in cortex and hippocampus

**Fig. 2:**
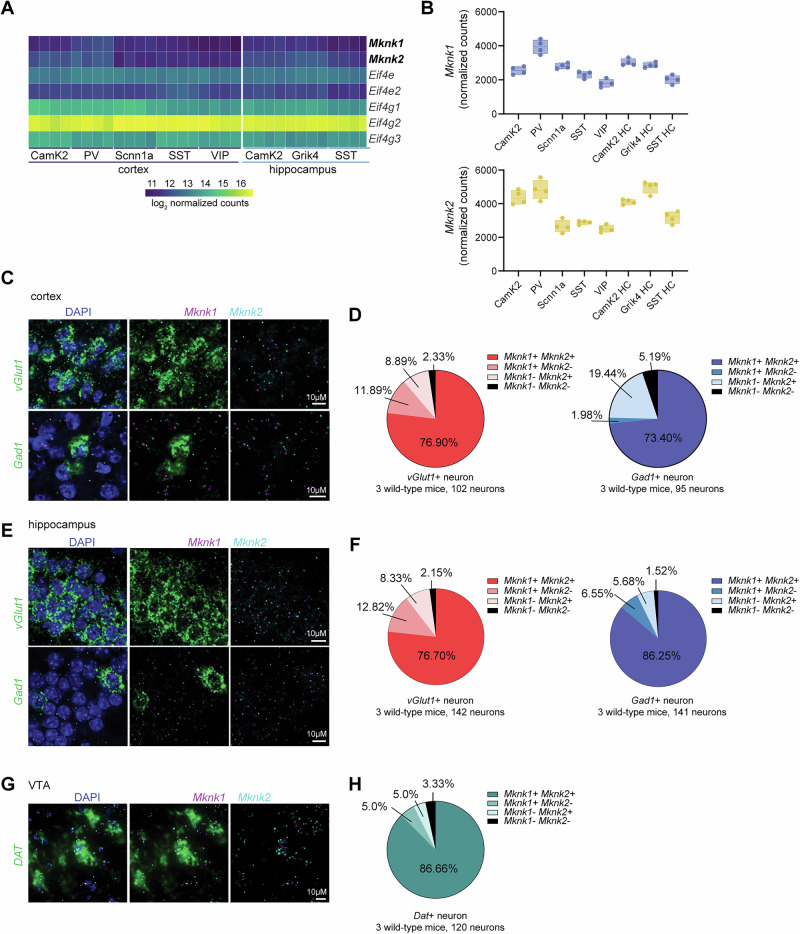
MNK1 and MNK2 have overlapping expression patterns in cortex and hippocampus. (**A-B**) Heatmap (**A**) and boxplot (**B**) of *Mknk1* and *Mknk2* across different neuronal populations in cortex and hippocampus. *Eif4e* and *Eif4g* isoforms are shown for comparison in the heatmap. The mRNA expression was measured using Ribo-TRAP sequencing from Furlanis et al. (2019). (**C, E**) Representative fluorescent in situ hybridization (FiSH) images in cortex (**C**) and hippocampus (**E**) using probes for *vGlut1* (green), *Gad1* (green), *Mknk1* (magenta), and *Mknk2* (cyan). DAPI is in blue. (**D, F**) Pie-chart showing the distribution of *Mknk1* and *Mknk2* in *vGlut1* (left) and *Gad1* (right) positive neurons in cortex (**D**) and (**F**) hippocampus. (**G**) Representative images of *Mknk1 (magenta)* and *Mknk2 (cyan)* expression in neurons expressing the dopamine transporter (DAT) (green) in the ventral tegmental area (VTA). (**H**) Pie chart showing the distribution of *Mknk1* and *Mknk2* in *DAT*-positive neurons in the VTA. The total number of neurons is listed under each pie chart. The percentage of each neuronal population is an average of three mice. Camk2=calcium/calmodulin-dependent protein kinase II positive neurons, PV=Parvalbumin-positive interneurons, Scnn1a=sodium channel, nonvoltage-gated 1α positive spiny stellate and star pyramid layer 4 (L4) neurons, SST=somatostatin-positive interneurons, VIP=vasointestinal peptide-positive interneurons, Grik4=glutamate receptor, ionotropic, kainate 4-positive interneurons, HC=hippocampus.

A possible explanation for the different behavioral phenotypes in the MNK1^KO^ and MNK2^KO^ mice is that the MNKs are expressed in distinct neuronal populations. Transcriptomic studies suggest that MNK1 and MNK2 are expressed throughout the brain in mice, humans, and pigs [12, 33, 51], with ribosome-bound *Mknk1* and *Mknk2* mRNA also detected in synaptic neuropil [34]. To further characterize their expression, we took advantage of published RNA-sequencing data from ribosome-bound mRNAs (RiboTRAP) from multiple neuronal cell types in the cortex and hippocampus [52]. *Mknk1* and *Mknk2* mRNAs were broadly expressed throughout all examined cell types, with slightly higher expression of *Mknk2* (Fig. 2A-B) [52]. Published single-cell sequencing data also suggest overlapping mRNA expression of *Mknk1* and *Mknk2* in multiple cell types in the amygdala [51]. To further assess MNK brain expression, we performed fluorescent in situ hybridization (FiSH) using probes against *Mknk1* and *Mknk2* in cortex, hippocampus, and the ventral tegmental area (VTA). In the cortex, we found that *Mknk1* and *Mknk2* were expressed in almost all neurons, with most excitatory (*vGlut1* positive) and inhibitory (*Gad1* positive) neurons expressing both *Mknk1* and *Mknk2* (Fig. 2C-D). A similar expression pattern was found in hippocampus (Fig. 2E-F) and in dopaminergic neurons in the VTA (Fig. 2G-H). Taken together, these results suggest that MNK1 and MNK2 are expressed in largely overlapping neuronal populations in several brain regions in mice.

### Proteomic analysis of MNK1 and MNK2 knockout mice show different effects on the synaptic proteome

**Fig. 3:**
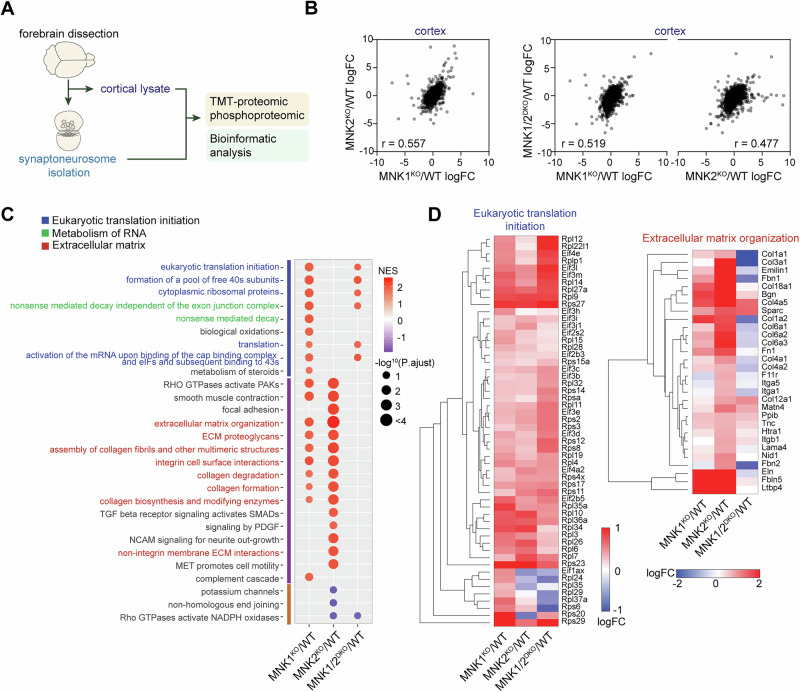
Proteomic analysis of MNK1 and MNK2 knockout mice. (**A**) Schematic of the experimental procedure. Cortex was dissected and synaptoneurosomes were isolated using the filter method. The whole homogenate was used as input for the cortical proteome. (**B**) Pearson correlation of the cortical proteome Log fold change (LogFC, relative to wild-type) in MNK1^KO^, MNK2^KO^, and MNK1/2^DKO^. (**C**) Dot plot of selected significantly enriched (FDR < 0.25) canonical pathways of Gene set enrichment analysis (GSEA) comparing MNK1^KO^, MNK2^KO,^ and MNK1/2^DKO^ cortical proteome relative to wild-type. Only significant pathways are shown per genotype. (**D**) Heatmap showing logFC of the core enrichment proteins in the eukaryotic translation initiation (left) and extracellular matrix organization (right) signaling pathways. WT=wild-type, NES=normalized enrichment score.

The specific behavioral phenotypes of MNK1 and MNK2 knockout mice suggest that the MNKs may regulate distinct aspects of neuronal translation. To start investigating how MNK1 and MNK2 affect protein expression, we performed tandem mass tag (TMT)-based mass spectrometry on the whole homogenate and isolated synaptoneurosomes from cortex of MNK1^KO^, MNK2^KO^, and MNK1/2^DKO^ mice compared to wild-type mice (Fig. 3A). In the whole homogenate from cortex, we found that loss of either MNK1, MNK2, or both kinases had a similar effect on the proteome, with a significant correlation in protein log fold change (logFC) relative to wild-type between all genotypes (Fig. 3B). Consistent with the proteomic profile of mice treated with an MNK inhibitor [14], few significantly differentially expressed proteins were identified in the cortical proteome for any genotype (adjusted p-value < 0.05, Supplementary Table 3). We used gene set enrichment analysis (GSEA) to identify molecular pathways with altered expression between genotypes (FDR < 0.25, Supplementary Table 3). Overall, loss of MNK1 or MNK2 affected both overlapping and distinct pathways in cortex (Fig. 3C-D, Supplementary Table 3). Hierarchical clustering identified two main clusters, with cluster 1 enriched in pathways related to translation and cluster 2 enriched in pathways associated with the extracellular matrix (Fig. 3D, Supplementary Table 3), consistent with previous studies using MNK1/2^DKO^ mice [15].

**Fig. 4:**
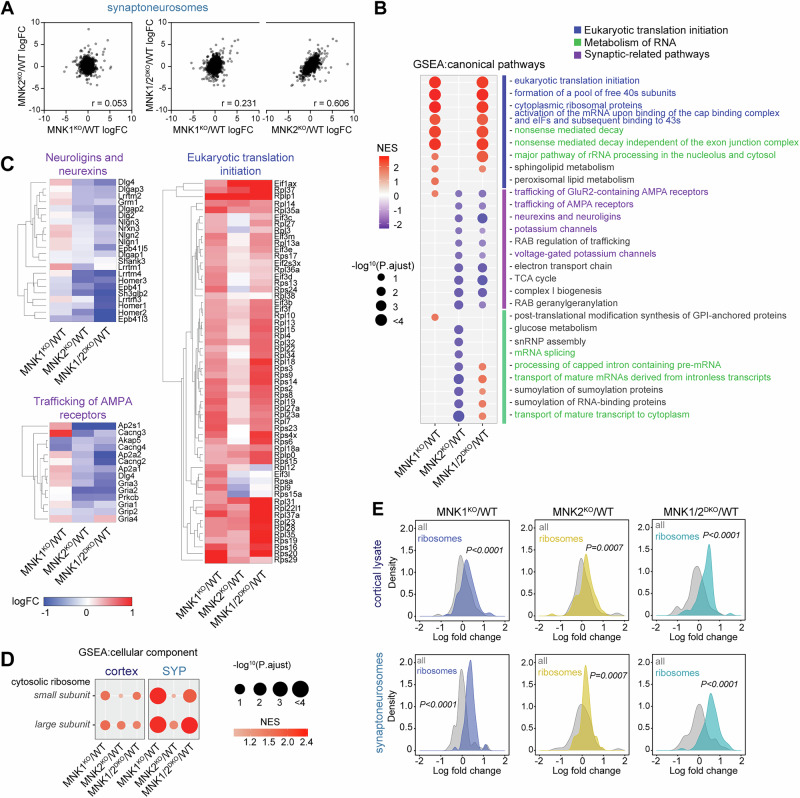
Distinct contribution of MNK1 and MNK2 to the synaptic proteome. (**A**) Pearson correlation of the synaptoneurosome proteome logFC relative to wild-type in MNK1^KO^ versus MNK2^KO^ (left), and of MNK1^KO^ and MNK2^KO^ versus MNK1/2^DKO^ (right). (**B**) Dot plot of selected significantly enriched (FDR < 0.25) canonical pathways of GSEA comparing MNK1^KO^, MNK2^KO^, and MNK1/2^DKO^ synaptoneurosome proteome to wild-type. Only significant pathways are shown for each genotype. (**C**) Heatmap showing logFC relative to wild-type of the core enrichment proteins in the neurexins and neuroligins, trafficking of AMPA receptors, and eukaryotic translation initiation signaling pathways. (**D**) GSEA of cytosolic ribosome cellular component GO term enrichment for MNK1^KO^, MNK2^KO,^ and MNK1/2^DKO^ relative to wild-type. (**E**) Density plots of logFC ribosomal protein abundance compared to all proteins in the cortical (top) and synaptoneurosome (bottom) proteome for all genotypes relative to wild-type. P-values in E were calculated using a two-sided Kolmogorov-Smirnov test.

Synaptic dysfunction is a hallmark of many neurodevelopmental conditions. Synaptic activity can induce eIF4E phosphorylation, and MNK1 has previously been implicated in synaptic translation [15, 17, 18]. Therefore, we next examined how knockout of the MNKs affected the synaptic proteome. We isolated synaptoneurosomes using a protocol that enriches both the pre-and postsynaptic compartments [5, 53], and confirmed enrichment by comparing the proteome of cortical homogenate to the synaptoneurosome fraction in wild-type animals (Supplementary Figure 4) [47, 54]. In stark contrast to the cortical proteome, comparing the protein LFC relative to wild-type in the synaptoneurosome fractions between the different genotypes showed a very low correlation between MNK1^KO^ and MNK2^KO^ mice (Fig. 4A, Supplementary Figure 5A). The protein expression of MNK1/2^DKO^ mice was highly correlated with mice lacking MNK2 but not MNK1, suggesting that the majority of altered protein expression in synaptoneurosomes from MNK1/2^DKO^ mice is driven by the loss of MNK2. Of note, the low correlation between MNK1^KO^ and MNK2^KO^ mice synaptoneurosomes appears to be driven by MNK2 deletion differentially affecting the cortical and synaptoneurosome proteome, whereas deletion of MNK1 causes similar proteomic changes in both fractions (Supplementary Figure 5A).

GSEA identified three major clusters of pathways with altered abundance (Fig. 4B). The first cluster consisted of pathways related to mRNA translation and RNA metabolism. These were highly enriched in MNK1^KO^ mice synaptoneurosomes, whereas synapse and RNA metabolism pathways in the second and third clusters were de-enriched in MNK2^KO^ mice (Fig. 4B-C, Supplementary Table 4). Examination of the core proteins accounting for the enrichment in the translation and RNA metabolism pathways in cluster one identified ribosomal proteins as the main group of proteins overexpressed in MNK1^KO^ mice (Fig. 4C), and a comparison between cortex and synaptoneurosomes showed that this enrichment was more pronounced at the synapse (Fig. 4D, Supplementary Table 4). To examine if this enrichment affected ribosomal proteins as a group or was specific to a subset of ribosomal proteins, we analyzed the expression of all ribosomal subunits identified in our dataset. We found a significant upregulation of both small and large ribosomal subunits that was more pronounced in synaptoneurosomes from mice lacking MNK1 (Fig. 4E). Increased expression of ribosomal proteins was also found in wild-type mice treated with an MNK inhibitor (Supplementary Figure 5B) [14], and in a previous proteomic dataset of de novo synthesized proteins, translation of ribosomal proteins was reduced in wild-type compared to MNK1 knockout primary neurons treated with BDNF, suggesting that MNK1 modulates ribosomal protein synthesis (Supplementary Figure 5C) [17]. Together, these results strongly suggest that MNK1 inhibition or deletion elevates ribosomal protein expression.

### Comparison of mRNA and protein reveals translational and transcriptional upregulation of ribosomal proteins in MNK1^KO^ mice

The differences in protein expression at the synapse of MNK1^KO^ and MNK2^KO^ mice could be caused by an altered abundance of local mRNAs, posttranscriptional modification, or changes in transport. To examine if mRNA abundance contributed to the proteomic differences, we performed mRNA sequencing (RNA-Seq) on isolated cortical synaptoneurosomes from MNK1^KO^, MNK2^KO^, and wild-type mice. We identified 637 mRNAs significantly altered in synaptoneurosomes from MNK1^KO^ mice and 112 from MNK2^KO^ mice (Supplementary Figure 6A-B, Supplementary Table 5), suggesting that loss of MNK1 has a larger effect on the synapse-enriched transcriptome compared to loss of MNK2. In agreement with the proteomic dataset, the mRNA log fold change relative to wild-type showed a low correlation between MNK1^KO^ and MNK2^KO^ mice (Supplementary Figure 6C), suggesting that loss of MNK1 or MNK2 also has a largely distinct effect on the synaptic transcriptome. There was also a low correlation between mRNA and protein expression in both MNK1 and MNK2 knockout mice (Supplementary Figure 6D-E).

**Fig. 5:**
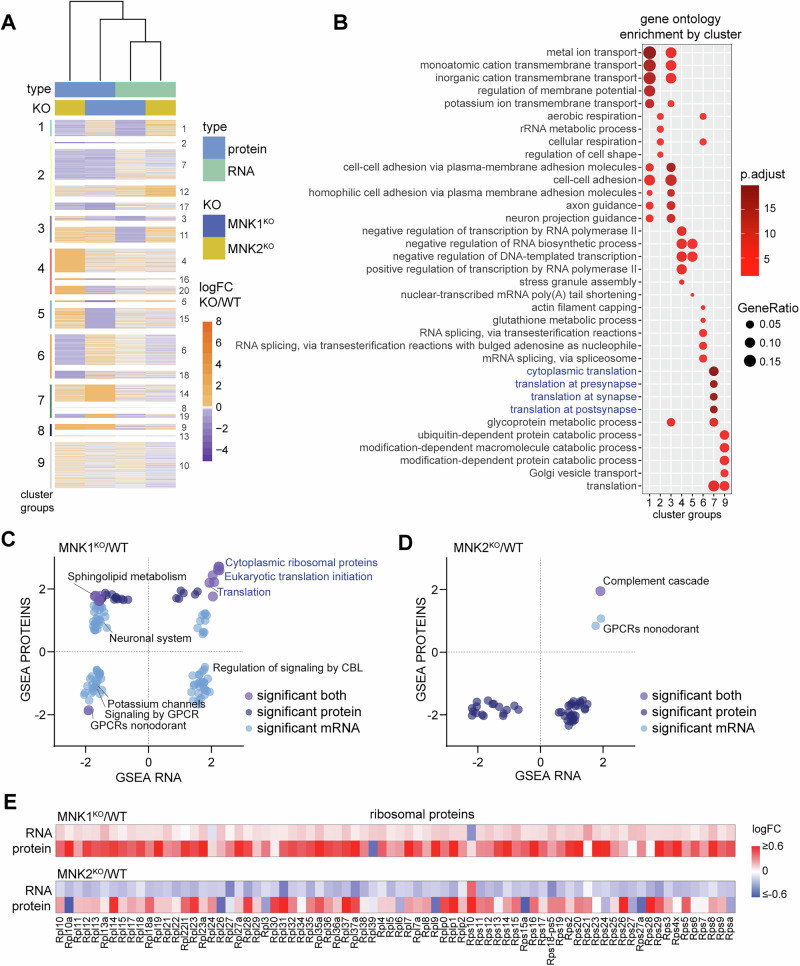
MNK1 and MNK2 knockout mice have different effects on the synaptic transcriptome. (**A**) Heatmap showing K-means clustering of protein and mRNA logFC values in MNK1^KO^ and MNK2^KO^ mice relative to wild-type. A total of 8294 genes are shown that are detected on both mRNA and protein levels. Groups of clusters with similar expression profiles are listed on the left, and the 20 k-means clusters on the right. (**B**) Gene ontology pathway enrichment analysis of the genes in each cluster group in (**A**) using all detected genes as a background. Top five significantly overrepresented GO terms per cluster are shown. (**C-D**) Overlap of GSEA canonical pathway terms significant in either the RNA-Seq or proteomic datasets or both for synaptoneurosome from (**C**) MNK1 and (**D**) MNK2 knockout mice. (**E**) Heatmap showing logFC of ribosomal subunit mRNA and protein from synaptoneurosomes from MNK1^KO^ mice (top) and MNK2^KO^ mice (bottom), relative to wild-type mice.

To examine the differences between protein and mRNA expression, we performed a multi-omics integration using the log fold change of genes identified in all datasets as input and K-means clustering to identify co-regulated groups of mRNAs and proteins. Unsupervised hierarchical clustering showed that mRNA expression changes were more similar in both knockouts compared to their proteome changes (Fig. 5A). The MNK2^KO^ proteome clustered separately from other samples, pointing towards a stronger effect of posttranscriptional regulation in MNK2^KO^ mice (Fig. 5A). To order the 20 identified clusters according to their expression patterns, we compared mean expression profiles of each cluster using Pearson correlations and then performed hierarchical clustering on the resulting distance matrix. This identified 9 groups of clusters, with most groups showing anti-correlated expression for mRNAs and proteins for one or both genotypes (Fig. 5A, Supplementary Table 5). We then performed gene ontology (GO) analysis on each group of clusters (Fig. 5B, Supplementary Table 5). Clusters 1 and 3 showed opposing mRNA expression profiles between MNK1^KO^ and MNK2^KO^ mice, with decreased mRNA expression in MNK1^KO^ mice but increased in MNK2^KO^ mice. Pathways related to ion transport, cell-cell adhesion, and axon guidance were significantly enriched in these clusters. The change in mRNA expression was anti-correlated with protein expression in most clusters, suggesting that the effect on protein expression is caused by altered translational regulation rather than mRNA abundance (Fig. 5A-B, Supplementary Figure 6F).

The multi-omic integration identified one cluster group (cluster 7) with strongly increased protein expression in MNK1^KO^ mice (Fig. 5A). GO analysis showed an enrichment of categories involved with cytoplasmic and synaptic translation in this cluster (Fig. 5B), consistent with our proteomic GSEA results. To further compare the transcriptomic and proteomic datasets, we performed GSEA on the transcriptomic dataset and compared gene sets significantly altered in either the transcriptome, proteome, or both (Fig. 5C-D, Supplementary Table 5). Interestingly, pathways related to translation were highly enriched for both protein and mRNA in synaptoneurosomes from MNK1^KO^ mice. In agreement with the proteomic dataset, the core enriched genes in these pathways were primarily ribosomal proteins, and analysis of individual ribosomal subunits confirmed an increase in mRNA and protein expression for almost all ribosomal subunits in synaptoneurosomes from MNK1^KO^ mice (Fig. 5E). Ribosomal proteins can be locally synthesized in neurites and incorporated into existing ribosomes, independent of ribosome biogenesis [55, 56]. Interestingly, we did not see an increase in ribosomal RNA (rRNA) in the cell body or synaptoneurosomes (Supplementary Figure 6G-J). These results suggest that although the majority of changes in protein expression in both MNK1 and MNK2 knockout mice is driven by posttranscriptional regulation, the increased expression of ribosomal proteins in MNK1^KO^ mice can be at least partially explained by an increase in ribosomal subunit mRNAs at the synapse.

### Absence of MNK1 and 2 affects protein synthesis rate and synaptic morphology

The behavioral, proteomic, and transcriptomic datasets suggest that MNK1 and MNK2 have specific functions in the nervous system and control distinct cellular processes at the synapse, with MNK1 affecting the expression of ribosomal proteins and MNK2 the expression of synaptic proteins. To determine if the observed changes in protein expression are associated with functional changes, we performed multiple experiments to assess neuronal protein synthesis and synaptic morphology.

**Fig. 6:**
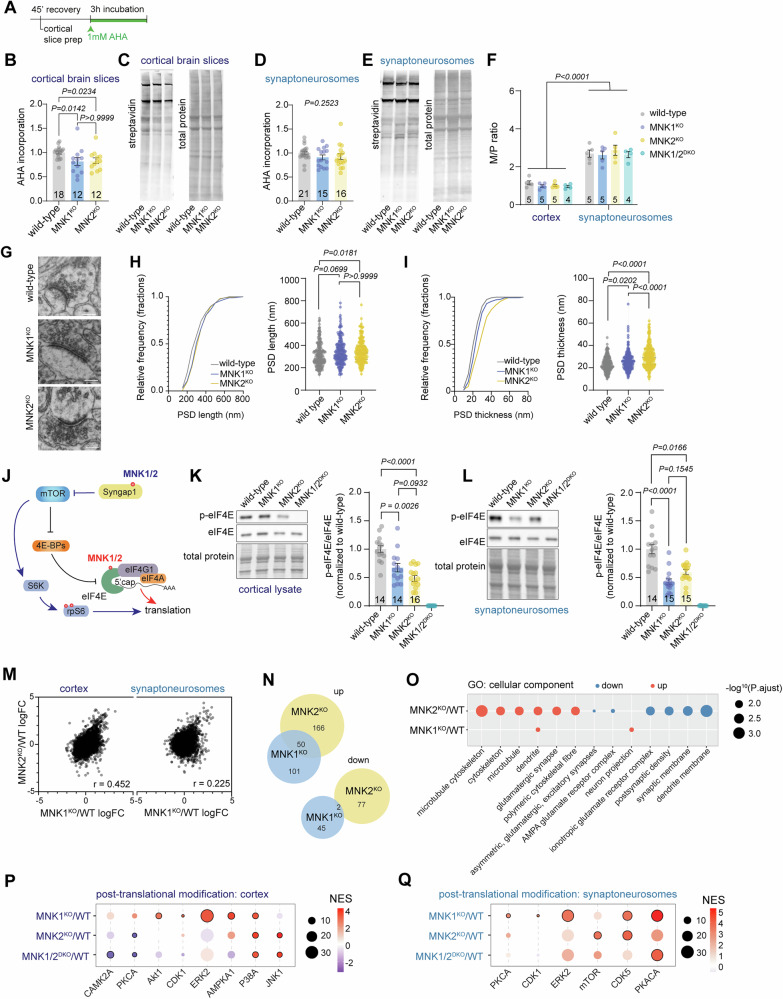
Phosphoproteomic characterization of MNK1 and MNK2 knockout mice. (**A**) Timeline of AHA incubation in brain slices. (**B**) Quantification and (**C**) representative image of AHA incorporation in cortical brain slices from wild-type, MNK1^KO,^ and MNK2^KO^ mice. (**D**) Quantification and (**E**) representative image of AHA incorporation in synaptoneurosomes from wild-type, MNK1^KO,^ and MNK2^KO^ mice. (**F**) Polysome profiling in cortical homogenate and synaptoneurosomes. Quantification of monosome/polysome ratio, representative traces are shown in Supplementary Figure 7A-B. (**G**) Representative electron microscopy images from wild-type, MNK1^KO^, and MNK2^KO^ mice. Scale bar: 0.2 µM. (**H-I**) Cumulative frequency (left) and violin plot (right) of postsynaptic density (PSD) length (**H**) and thickness (**I**). Number of synapses: wild-type n = 165 (2 mice), MNK1^KO^ n = 178 (3 mice), MNK2^KO^ n = 157 (3 mice). (**J**) Schematic of MNK1/2 regulation of mRNA translation via phosphorylation of Syngap1 (blue) and eIF4E (red). (**K-L**) Representative western blot (left) and quantification (right) of p-eIF4E compared to total eIF4E from (**K**) cortical lysates and (**L**) synaptoneurosomes from wild-type, MNK1^KO^, and MNK2^KO^ mice. MNK1/2^DKO^ mice are included for validation. (**M**) Alterations in MNK1^KO^ and MNK2^KO^ cortical and synaptoneurosomes phosphoproteome relative to wild-type. r was determined by Pearson correlation. (**N**) Venn diagram showing the overlap of phosphosites increased or decreased (LFC > 1.2 or LFC < –1.2 compared to wild-type) in synaptoneurosome phosphoproteome from MNK1^KO^ and MNK2^KO^ mice. (**O**) Bubble plot of cellular component GO terms enriched in MNK1^KO^ and MNK2^KO^ synaptoneurosome phosphoproteome. Red: increased, blue: decreased. (**P-Q**) Bubble plot of selected phosphosite-specific signatures in cortex (**P**) and synaptoneurosomes (**Q**) as determined by PTM-SEA. Significantly enriched kinase signatures (FDR < 0.05) are marked with a black circle, and the size corresponds to the number of observed phosphosites. All error bars are s.e.m. Significance was determined by Kruskal-Wallis test followed by Dunn’s multiple comparison test for B, D, H, I, L, and one-way ANOVA followed by Tukey’s multiple comparison test for F and K.

First, we examined if the rate of protein synthesis was altered in mice lacking MNK1 or MNK2. MNK1/2^DKO^ mice have no change in protein synthesis rate [11], but acute inhibition of the MNKs reduces the rate of protein synthesis in neurons [14]. We used incorporation of the non-canonical amino acid azidohomoalanine (AHA) to examine how the loss of MNK1 or MNK2 affected protein synthesis in cortical brain slices and synaptoneurosomes isolated from brain slices and found a small but significant reduction of translation rate in both MNK1 and MNK2 knockout mice in cortex (Fig. 6A-C), but not in synaptoneurosomes (Fig. 6D-E). We further examined protein synthesis using polysome profiling to estimate the degree of mRNA loading onto monosomes and polysomes in cortex and synaptoneurosomes. As previously reported [7], we found a large increase in the monosome/polysome (M/P) ratio in synaptoneurosomes compared to cortical homogenate, but there was no change in M/P ratio between the genotypes (Fig. 6F, Supplementary Figure 7A-B). To explore whether the deletion of MNK1 or MNK2 affects synaptic structure, we used electron microscopy. Compared to control animals, deletion of MNK1 and MNK2 caused a small but significant increase in the length and thickness of postsynaptic densities in the somatosensory cortex (Fig. 6G-I). Consistent with the proteomic data, these changes were more pronounced in the MNK2^KO^ mice, particularly the increase in PSD thickness (Fig. 6I).

### MNK1 and MNK2 have specific effects on the neuronal phosphoproteome

To examine potential mechanisms of how the MNKs exert their function, we first focused on the activity of their substrates. The MNKs can regulate neuronal translation via phosphorylation of eIF4E or Syngap1 [11, 15] (Fig. 6J). Mice with a mutation that prevents eIF4E phosphorylation show increased translation of ribosomal proteins [57], suggesting that MNK1-dependent eIF4E phosphorylation may regulate ribosomal protein expression. However, we observed no significant difference in eIF4E phosphorylation in cortical lysate or synaptoneurosomes in MNK1 and MNK2 knockout mice (Fig. 6K-L, Supplementary Figure 7C-D). As expected, no eIF4E phosphorylation was seen in the MNK1/2^DKO^ mice (Fig. 6K-L, Supplementary Figure 7C-D), and no difference was found in eIF4E protein levels (Supplementary Figure 7E-F). MNK1/2 can phosphorylate Syngap1 on S788, which promotes protein synthesis and increases phosphorylation of ribosomal protein S6 (rpS6) [15]. We found a decrease in rpS6 phosphorylation in cortical lysate in MNK1^KO^ mice and in synaptoneurosomes from both MNK1^KO^ and MNK2^KO^ mice (Supplementary Figure 7G-H). Phosphorylation of the mTOR substrate 4E-binding protein1 (4EBP1) was unaffected (Supplementary Figure 7I), and there was no difference in the upstream signaling pathway ERK1/2 (Supplementary Figure 7J-K). It is possible that a small but significant decrease in MNK1 protein expression in cortex from MNK2^KO^ mice affects these results (Supplementary Figure 7L-M). None of the antibodies we tested were specific for MNK2, and neither MNK1 nor MNK2 was detected in the proteome from either cortex or synaptoneurosomes. Therefore, we could not clarify whether MNK2 protein expression was similarly altered in MNK1^KO^ mice. Of note, the mRNA expression of *Mnk1* and *Mnk2* was not significantly altered in synaptoneurosomes from MNK2^KO^ and MNK1^KO^ mice, respectively (Supplementary Table 5).

Next, we performed phosphoproteomics to identify possible differences in other signaling pathways (Supplementary Table 6). Similar to the proteomic dataset, there was a high correlation between the phosphosite abundance changes relative to wild-type between MNK1^KO^ and MNK2^KO^ mice in the cortical homogenate but not in synaptoneurosomes (Fig. 6M, Supplementary Figure 8A). To determine which cellular functions were affected by MNK1 or MNK2 deletion, we performed a gene ontology (GO) analysis of increased and decreased phosphosites. Using a cutoff value of LFC > 1.2 or LFC < –1.2, we found multiple altered phosphosites, most of which were unique for each genotype (Fig. 6N, Supplementary Table 6). Interestingly, proteins related to synaptic function, particularly synaptic membranes, were overrepresented in proteins with decreased phosphorylation in MNK2^KO^ mice, whereas phosphorylation of proteins associated with the microtubule and cytoskeleton was increased (Fig. 6O). These results are consistent with the proteomic dataset and suggest that MNK2 deletion causes a decrease in both synaptic protein expression and phosphorylation, a change not seen in MNK1^KO^ mice.

To further explore how the MNKs affect signaling pathways, we performed a post-translational modifications signature enrichment analysis (PTM-SEA). We found several pathways significantly altered in both cortex and synaptoneurosomes (Fig. 6P-Q, Supplementary Figure 8B, Supplementary Table 6), including a decrease in Camk2a signaling, previously identified in synaptosomes from MNK1/2^DKO^ mice [15]. Hierarchical clustering showed that pathway changes were more similar between the cortical and synaptic fractions in MNK1^KO^ mice compared to MNK2^KO^ mice. The cortical fraction from MNK2^KO^ and MNK1/2^KO^ clustered separately from other samples, pointing to a different role for MNK2 depending on location (Supplementary Figure 8B). We focused on the synaptoneurosomes, where 4 PTM pathways were significantly altered in MNK1^KO^ mice and 2 in MNK2^KO^ mice. These pathways included an increase in CDK1 and cAMP-dependent protein kinase catalytic subunit alpha (PKACA) signaling in MNK1^KO^ mice, and an increase in mTOR signaling in MNK2^KO^ mice (Fig. 6Q, Supplementary Figure 8C). Together, these results suggest that MNK1 and MNK2 regulate distinct signaling pathways at the synapse.

## Discussion

To further develop the MNKs as drug targets, it is essential to better understand each kinase’s specific role in the nervous system. Using a multi-omic approach combined with detailed behavioral analysis, our study provides novel mechanistic insight into the isoform-specific function of the MNKs in the brain. We demonstrate that loss of MNK1 and MNK2 differentially affects the synaptic proteome and causes distinct social and cognitive behavioral phenotypes (Supplementary Figure 8D). Our results add to the numerous studies that suggest a degree of functional specification for the MNK proteins [17, 26, 29, 30], and indicate that it may be preferential to target each kinase individually.

Comparing the proteome between the whole cortex and the synaptic compartment allowed us to examine the location-specific effects of MNK deletion. We found that MNK1 and MNK2 have partially overlapping functions in cortex but distinct roles at the synapse, and that this difference is driven by a location-specific effect of MNK2. Our data indicate that both MNK1 and MNK2 are active in the somatic and synaptic compartments, suggesting that their divergent roles are not due to spatial segregation but rather to context-dependent regulation or substrate availability. In human cells, MNK2 is alternatively spliced into two isoforms with somewhat specialized functions and subcellular localization [58], and it may be that also in rodents, *Mknk2* splice isoforms localize to distinct compartments. Alternatively, MNK2 may engage distinct upstream activators or downstream substrates depending on its subcellular location.

Interestingly, deletion of either MNK1 or MNK2 had similar effects on eIF4E phosphorylation in the synaptic compartment, suggesting that altered steady-state phosphorylation of eIF4E is not the cause of the distinct phenotypes in MNK1 and MNK2 knockout mice. This finding was somewhat unexpected, given previous reports that MNK1 is more activity-dependent, while MNK2 has higher constitutive activity. One possible explanation is compensatory regulation between the kinases. Supporting this, we observed a slight increase in MNK1 protein levels in the synaptic fraction of MNK2^KO^ mice, which could buffer eIF4E phosphorylation in the absence of MNK2. However, since our results suggest distinct synaptic functions of MNK1 and MNK2, any compensation is likely limited. How phosphorylation of eIF4E impacts translation remains incompletely understood, but evidence from multiple studies suggests that it modulates translation in a context and cell-type-specific manner [17–19, 59]. Therefore, even in the absence of a baseline effect, it is possible that the differences seen in MNK1 and MNK2 knockout mice are at least partially driven by differences in how the MNKs regulate activity-dependent eIF4E phosphorylation, possibly in a stimulus-specific manner.

MNK inhibition has previously been shown to alter ribosomal protein expression [14, 15, 57, 59], and we here identify MNK1 as the kinase responsible for this change. The increase in ribosomal protein expression is likely at least partially driven by reduced eIF4E phosphorylation, as increased translation of ribosomal proteins has previously been found in eIF4E phosphorylation mutant mice and cells treated with MNK inhibitors [57, 59]. Interestingly, the shift in ribosomal protein expression was more pronounced at the synapse, suggesting the intriguing possibility that MNK1 may be of particular importance for synaptic translation. The increased abundance of ribosomal proteins at the synapse is supported by the overexpression of ribosomal subunit mRNAs in the synaptic fraction of MNK1^KO^ mice, which suggests that the increase in ribosomal proteins is caused by on-site translation rather than a shift in ribosomal protein stability.

The change in ribosomal protein expression is coupled with altered social and object memory in MNK1^KO^ mice and altered spatial memory in MNK1/2^DKO^ mice [15]. These results are consistent with previous work showing that altered ribosome expression is linked to memory dysfunction and altered long-term depression (LTD) [14, 60], and add to a growing body of research suggesting that precise regulation of local ribosomal protein expression may be necessary to support synaptic plasticity and memory [4, 56, 61]. Indeed, the lack of cognitive behavioral phenotype in MNK2^KO^ mice, despite the reduction of synaptic proteins, could be related to the slight increase in MNK1 expression and unchanged levels of synaptic ribosomal proteins in these mice. However, it is important to note that although our data support a role for synaptic MNK function in shaping behavior, MNKs are expressed in most cell types, and we cannot exclude the possibility that developmental effects or non-neuronal mechanisms contribute to the observed phenotypes.

Since we did not detect changes in overall synaptic translation in either genotype, the functional impact of increased ribosomal protein expression remains unclear. Mice with a phospho-deficient eIF4E also exhibit increased translation of ribosomal proteins without a change in translation rate [13, 57], suggesting that excess levels of ribosomal proteins do not always affect general translation. Instead, altered ribosome levels have been linked to a length-dependent shift in translation [60, 62]. It is therefore possible that the excess of ribosomal proteins found in MNK1^KO^ mice affects what is being translated rather than the overall translation rate. Locally synthesized ribosomal subunits can be incorporated into cytosolic ribosomes in neurites, where they may act to repair or modify ribosomal function [55, 56]. Since we did not detect any changes in rRNA, it may be that the increase in ribosomal proteins is not caused by de novo assembly of ribosomes, but rather by local synthesis of ribosomal subunits to modify or maintain synaptic ribosomes. Another possibility is that there is increased stalling of ribosomes in the MNK1^KO^ mice. MNK-dependent eIF4E phosphorylation has been shown to promote translation via facilitating the release of the CYFIP1/FMRP complex from the 5’-mRNA cap [17–19]. However, the MNK’s impact on translation is complex and is not only dependent on their kinase activity. For example, MNK1 was recently found to interact directly with ribosomal proteins and members of the eIF complex [31], whereas MNK2 can negatively regulate translation via direct interaction with eIF4G and inhibition of mTOR [32]. Although we did not identify any changes in eIF4G phosphorylation in our dataset, our pathway analysis identified an increase in mTOR signaling in mice lacking MNK2, including increased phosphorylation of mTORC1 component regulatory-associated protein of mTOR (Raptor) and Larp1. As mTOR and Larp1 are key regulators of ribosome production [63], we cannot rule out that MNK2 can act as an enhancer of ribosome protein expression via interaction with the mTOR pathway [63], perhaps even via direct interaction with Raptor [31]. This could explain the slight reduction of ribosomal subunit mRNAs in MNK2^KO^ mice, although more work is needed to test this.

Taken together, our results suggest a model where MNK1 regulates ribosomal protein expression via eIF4E phosphorylation at the synapse, whereas MNK2 regulates the translation of a pool of mRNAs that include synaptic proteins via other, possible mTOR-dependent mechanisms, although the exact mechanisms remain to be determined. This model is supported by the fact that both ribosomal and synaptic proteins are altered in the MNK1/2^DKO^ mice. Overall, our work may help clarify each kinase’s individual contribution to the therapeutic effects of MNK inhibitors and suggests that targeting MNK1 or MNK2 could differentially affect synaptic function.

## Supplementary information

Supplementary Information

Supplementary Table 1

Supplementary Table 2

Supplementary Table 3

Supplementary Table 4

Supplementary Table 5

Supplementary Table 6

Supplementary Table 7

## Data Availability

The mass spectrometry proteomics data have been deposited to the ProteomeXchange Consortium via the PRIDE [64] partner repository with identifier PXD058409. The RNA sequencing data have been deposited in the EBI repository with the accession number E-MTAB-16276.
