# Supplementary material for: Distinct roles for MNK1 and MNK2 in social and cognitive behavior through kinase-specific regulation of the synaptic proteome and phosphoproteome
Source: Mol Psychiatry. 2026 Feb 13;31(6):3446–61. doi: 10.1038/s41380-026-03483-w (PMC13190234; doi:10.1038/s41380-026-03483-w)
Supplement: Supplementary file 1 — Supplementary Information [file 41380_2026_3483_MOESM1_ESM.pdf]

Supplementary Information

Supplementary tables:

Supplementary Table 1
Statistical analysis of sex differences for the behavioral phenotyping (PDF file).

Supplementary Table 2
List of the behavioral categories used for merged behavioral phenotyping (Excel file).

Supplementary Table 3
Detailed data from the cortical proteome from $MNK1^{KO}$, $MNK2^{KO}$, $MNK1/2^{DKO}$, and wild-type mice (Excel file). Sheet 1 lists normalized protein expression, logFC, and P-values compared to wild-type. Sheet 2 contains GSEA results. Sheet 3 contains the results from the cluster analysis of significant pathways identified in the GSEA.

Supplementary Table 4
Detailed data from the synaptoneurosome proteome from $MNK1^{KO}$, $MNK2^{KO}$, $MNK1/2^{DKO}$, and wild-type mice (Excel file). Sheet 1 lists normalized protein expression, logFC, and P-values compared to wild-type. Sheet 2 contains GSEA results, Sheet 3 the cluster analysis, and Sheet 4 the GSEA of cellular component focusing on ribosomal protein categories.

Supplementary Table 5
Detailed data from the synaptoneurosome mRNA-sequencing from $MNK1^{KO}$, $MNK2^{KO}$, and wild-type mice (Excel file). Sheet 1 lists expression values, logFC, and p-values relative to wild-type, Sheet 2 lists the full result from the k-means cluster, and Sheets 2-3 contain the results from the GSEA.

Supplementary Table 6
Detailed data from the phosphoproteomic analysis (Excel file). Sheets 1-2 list expression values, logFC, and p-values relative to wild-type for cortex and synaptoneurosomes, Sheet 3 contains the top altered phosphosites, Sheet 3 the GO cellular component results, and Sheets 4-5 the PTM-SEA results.

Supplementary Table 7
Detailed statistical information for all figures (PDF file).

Supplementary figures:

Supplementary Figure 1
Detailed social phenotyping using DeepOF shows social phenotypes in $MNK1^{KO}$ and $MNK2^{KO}$ mice.

Supplementary Figure 2
Sex-specific analysis of the behavioral phenotypes in $MNK1^{KO}$ and $MNK2^{KO}$ mice

Supplementary Figure 3
Behavioral phenotyping of $MNK1/2^{DKO}$ mice.

Supplementary Figure 4
Validation of the synaptoneurosome enrichment.

Supplementary Figure 5
Comparison between the cortical and synaptic proteome shows site-specific effects of MNK2 deletion.

Supplementary Figure 6
Knockout of MNK1 or MNK2 has different effects on the synaptic transcriptome.

Supplementary Figure 7
Effect of MNK1 and MNK2 deletion on protein abundance and phosphorylation levels in pathways related to protein synthesis.

Supplementary Figure 8
Effect of MNK1 and MNK2 deletion on the cortical and synaptic phosphoproteome.

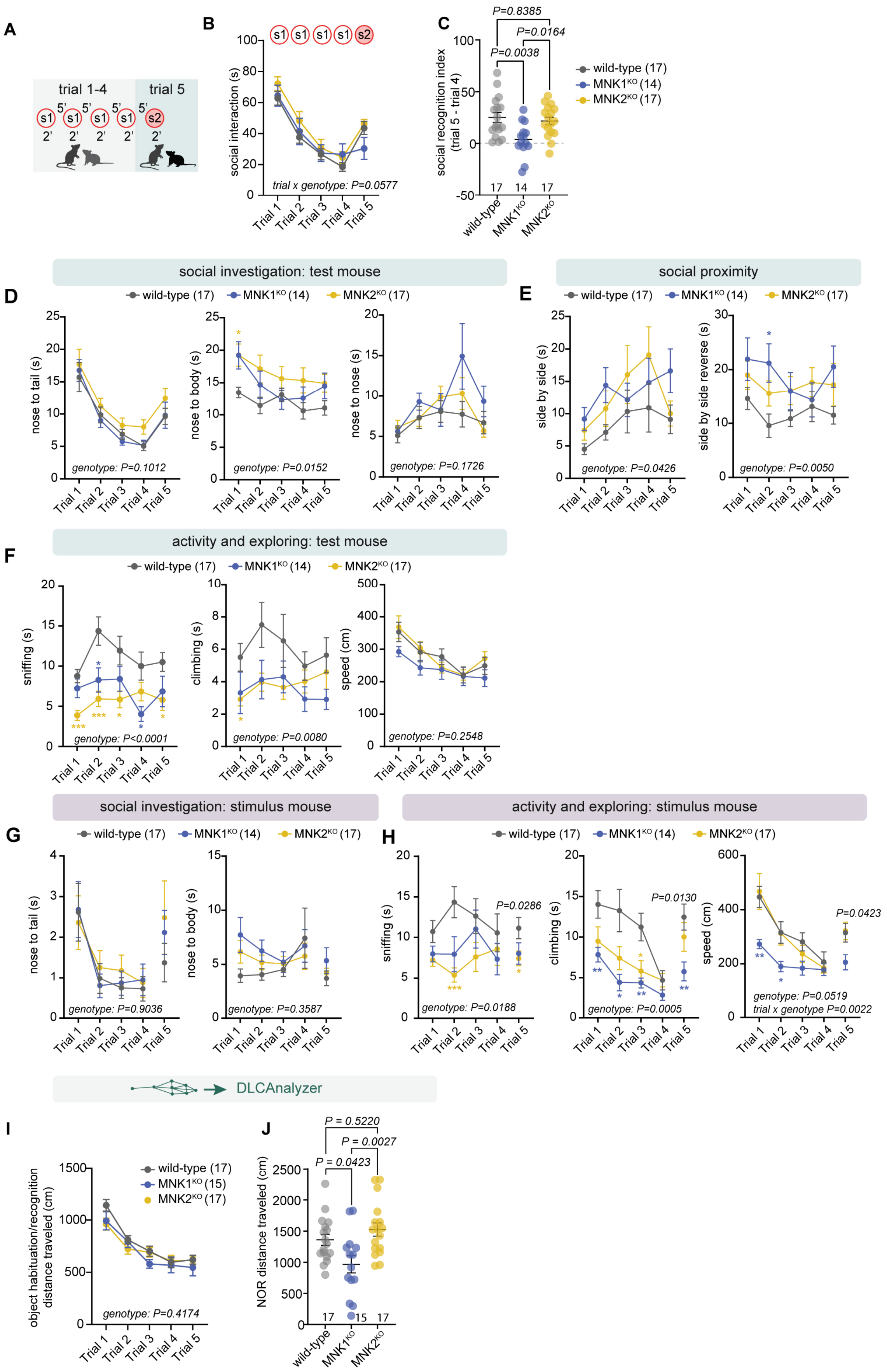
A
trial 1-4
trial 5
B
social interaction (s)
trial x genotype: P=0.0577
C
social recognition index (trial 5 - trial 4)
P=0.8385
P=0.0164
P=0.0038
wild-type (17)
MNK1KO (14)
MNK2KO (17)
social investigation: test mouse
D
nose to tail (s)
genotype: P=0.1012
nose to body (s)
genotype: P=0.0152
nose to nose (s)
genotype: P=0.1726
social proximity
E
side by side (s)
genotype: P=0.0426
side by side reverse (s)
genotype: P=0.0050
activity and exploring: test mouse
F
sniffing (s)
genotype: P<0.0001
climbing (s)
genotype: P=0.0080
speed (cm)
genotype: P=0.2548
social investigation: stimulus mouse
G
nose to tail (s)
genotype: P=0.9036
nose to body (s)
genotype: P=0.3587
activity and exploring: stimulus mouse
H
sniffing (s)
P=0.0286
genotype: P=0.0188
climbing (s)
P=0.0130
genotype: P=0.0005
speed (cm)
P=0.0423
genotype: P=0.0519
trial x genotype P=0.0022
DLCAnalyzer
I
object habituation/recognition distance traveled (cm)
MNK1KO (15)
genotype: P=0.4174
J
NOR distance traveled (cm)
P = 0.5220
P = 0.0027
P = 0.0423

Supplementary Figure 1. Detailed social phenotyping using DeepOF shows social phenotypes in MNK1^{KO} and MNK2^{KO} mice.

(A) Schematics of the social habituation/recognition test. (B-C) Mean social interaction time (B) and recognition index (C) in the social habituation/recognition test for wild-type, MNK1^{KO,} and MNK2^{KO} mice. s1=first social stimulus, s2=second social stimulus. (D-H) DeepOF analysis of social and individual behaviors in the five-trial social habituation/recognition test in wild-type, MNK1^{KO}, and MNK2^{KO} mice. (D) Social interaction from the test mouse towards the stimulus mouse covering nose to tail (left), nose to body (middle), and nose to nose (right). (E) Passive social behavior where the mice are in close proximity without directly interacting, either facing the same direction (side by side, left), or facing the opposite direction (side by side reverse, right). (F) Individual behavior of the test mouse, showing time sniffing the wall (left), time spent climbing/rearing on the wall (middle) and total speed during the trial (right). (G) Social interaction of the stimulus mouse towards the test mouse covering nose to tail (left) and nose to body (right). (H) Individual behavior of the stimulus mouse showing time sniffing the wall (left), time spent climbing/rearing on the wall (middle), and total speed during the trial (right). (I-J) Distance traveled during the object habituation/recognition test (I) and novel object recognition test (J). Animals were tracked with DeepLabCut and distance traveled analyzed using DLCAnalyzer. Error bars show s.e.m. Significance was determined using two-way RM ANOVA for B, I, one-way ANOVA followed by Tukey’s post-hoc test for C, a mixed-effect model followed by Tukey's post-hoc test for D-F, RM two-way ANOVA followed by Tukey's post-hoc test (trial 1-4) and Kruskal-Wallis test followed by Dunn's multiple comparison test (trial 5) for G-H, and one-way ANOVA followed by Tukey's post-hoc test for J.

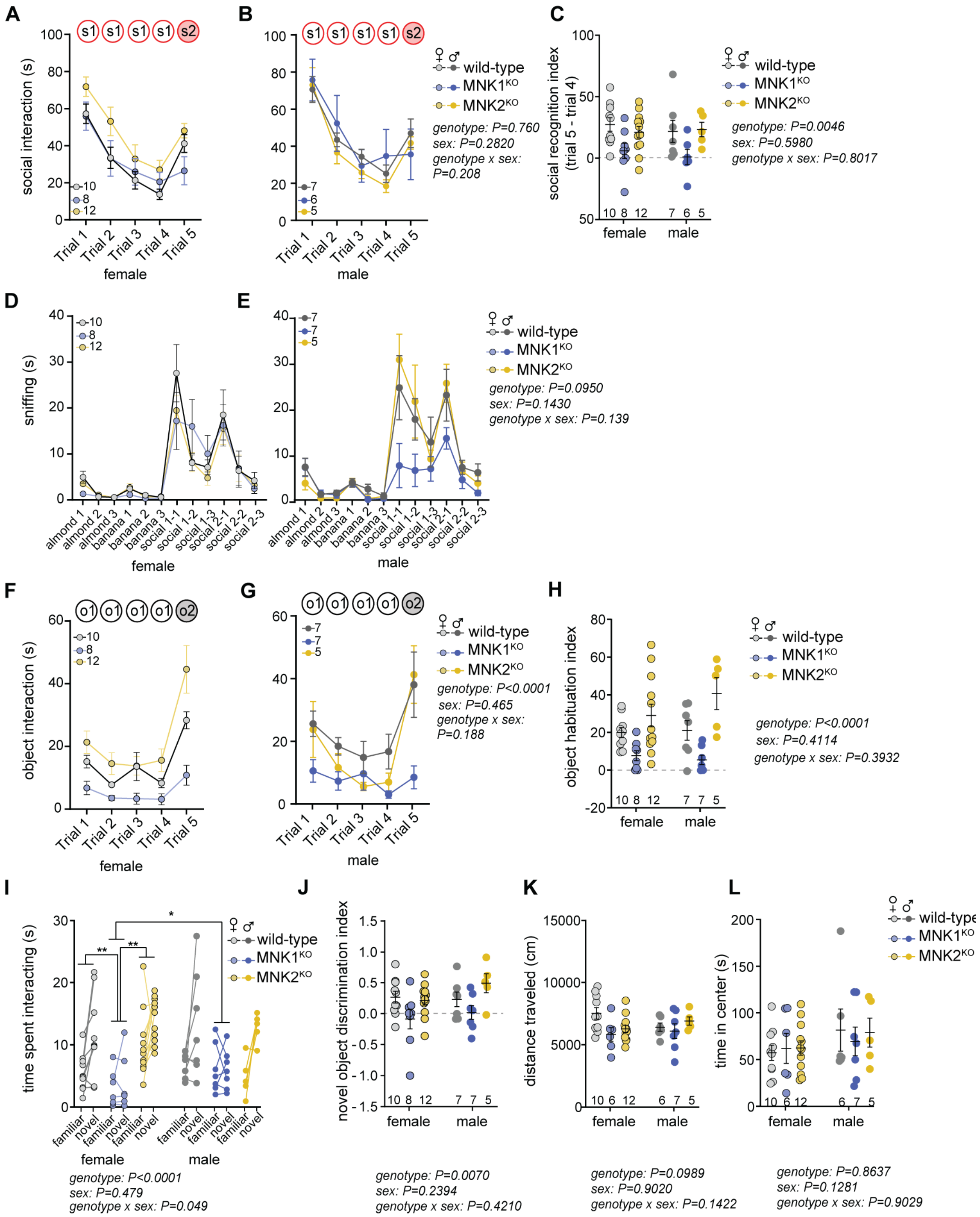

Supplementary Figure 2. Sex-specific analysis of the behavioral phenotypes in MNK1^{KO} and MNK2^{KO} mice.

(A-C) Mean social interaction time for females (A) and males (B), and recognition index (C) in the social habituation/recognition test in wild-type, MNK1^{KO}, and MNK2^{KO} mice. s1=first social stimulus, s2=second social stimulus. (D-E) Time sniffing for females (D) and males (E) in the social olfaction habituation test. (F-H) Mean object interaction time for females (E) and males (G), and recognition index (H) in the object habituation/recognition test. o1=first object stimulus, o2=second object stimulus. (I-J) Time spent interacting with the familiar and novel

object (I) and novel object discrimination index (J) in the novel object recognition test in male and female mice. (K-L) Total distance traveled (K) and time spent in center (L) in the open field test. Error bars show s.e.m. P values: *<0.5, **<0.01, ***<0.001 relative to wild-type. Significance was determined by three-way RM ANOVA followed by Bonferroni's post-hoc test for A, B, D, E, F, G, I, and two-way ANOVA followed by Tukey's post-hoc test for C, H, J, K, L.

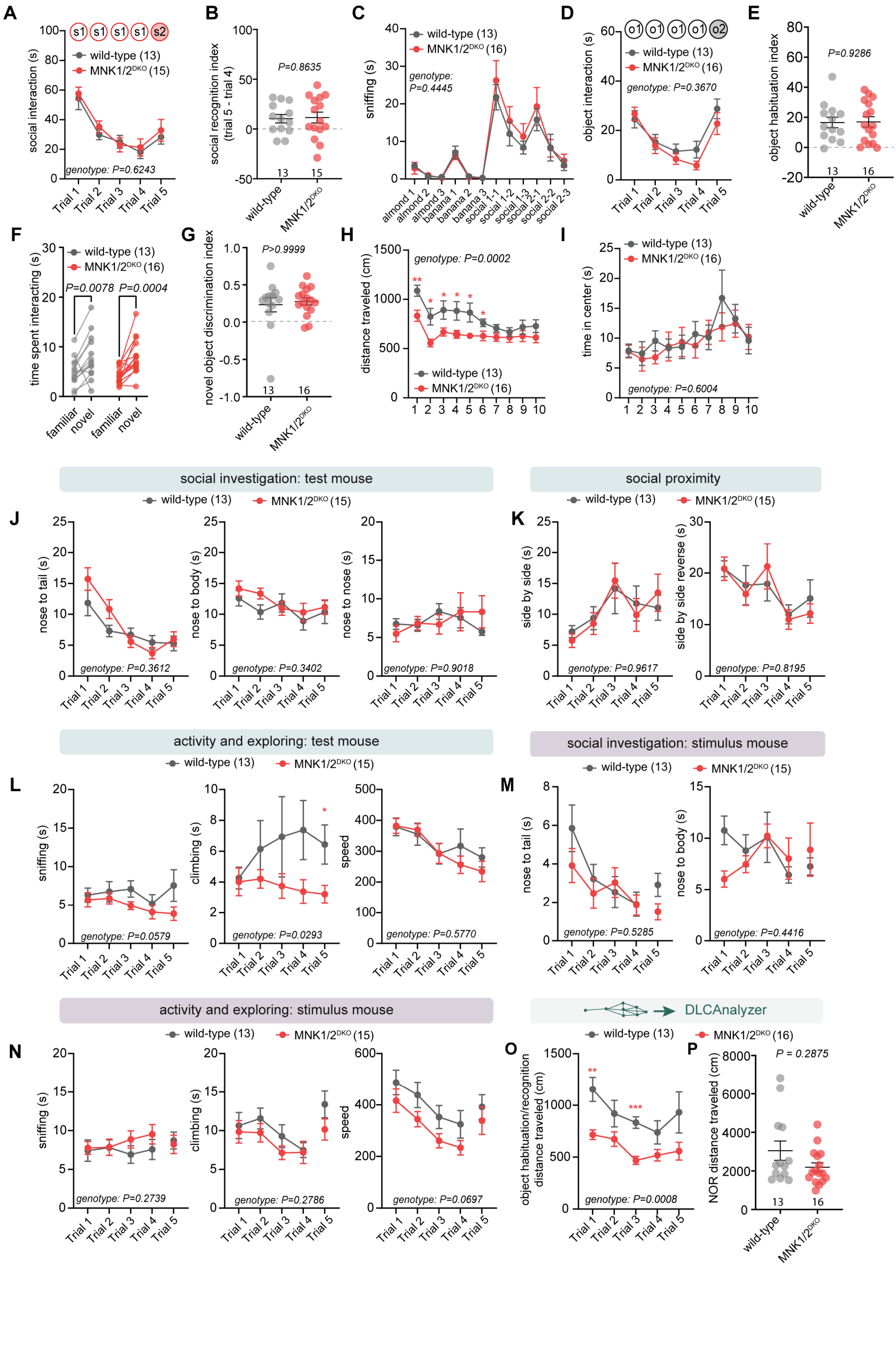

A
B
C
D
E
F
G
H
I
J
K
L
M
N
O
P
wild-type (13)
MNK1/2DKO (15)
MNK1/2DKO (16)
genotype: P=0.6243
P=0.8635
genotype: P=0.4445
genotype: P=0.3670
P=0.9286
P=0.0078
P=0.0004
P>0.9999
genotype: P=0.0002
genotype: P=0.6004
social investigation: test mouse
social proximity
genotype: P=0.3612
genotype: P=0.3402
genotype: P=0.9018
genotype: P=0.9617
genotype: P=0.8195
activity and exploring: test mouse
social investigation: stimulus mouse
genotype: P=0.0579
genotype: P=0.0293
genotype: P=0.5770
genotype: P=0.5285
genotype: P=0.4416
activity and exploring: stimulus mouse
DLCAnalyzer
genotype: P=0.2739
genotype: P=0.2786
genotype: P=0.0697
genotype: P=0.0008
P = 0.2875

Supplementary Figure 3. Behavioral phenotyping of MNK1/2^{DKO} mice.
(A-B) Mean social interaction time (A) and recognition index (B) in the social habituation/recognition test for wild-type and MNK1/2^{DKO} mice. s1=first social stimulus, s2=second social stimulus. (C) Time spent sniffing each odor in the social olfaction habituation test. (D-E) Mean object interaction time (D) and recognition index (E) in the object habituation/recognition test. o1=first object, o2=second object. (F-G) Time spent interacting with the familiar and novel object (F) and novel object discrimination index (G) in the novel object recognition test. (H-I) Distance traveled (H) and time spent in center (I) per minute in the open field test. (J-N) DeepOF analysis of social and individual behaviors in the five-trial social habituation/recognition test in wild-type and MNK1/2^{DKO} mice. (J) Social interaction from the test mouse towards the stimulus partner covering nose to tail (left), nose to body (middle), and nose to nose (right). (K) Passive social behavior facing the same direction (side by side, left), or facing the opposite direction (side by side reverse, right). (L) Individual behavior of the test mouse, showing time sniffing the wall (left), time spent climbing/rearing on the wall (middle), and total speed during the trial (right). (M) Social interaction of the stimulus mouse towards the test mouse, covering nose to tail (left) and nose to body (right). (N) Individual behavior of the stimulus mouse showing time sniffing the wall (left), time spent climbing/rearing on the wall (middle), and total speed during the trial (right). (O-P) Distance traveled during the object habituation/recognition test (O), and novel object recognition test (P). Error bars show s.e.m. P values: *<0.5, **<0.01, ***<0.001 relative to wild-type. Significance was determined by two-way RM ANOVA followed by Tukey’s post-hoc test for A, C, D, H, I, J, K, L or Šídák’s post-hoc test for F, unpaired t-test for B, E, Mann-Whitney test for G, P, RM two-way ANOVA followed by Tukey's post-hoc test (trial 1-4) and Mann Whitney test (trial 5) for M, RM two-way ANOVA followed by Tukey's post-hoc test (trial 1-4) and unpaired t-test test (trial 5) for N, and Mixed-effect model followed by Tukey’s post-hoc test for O.

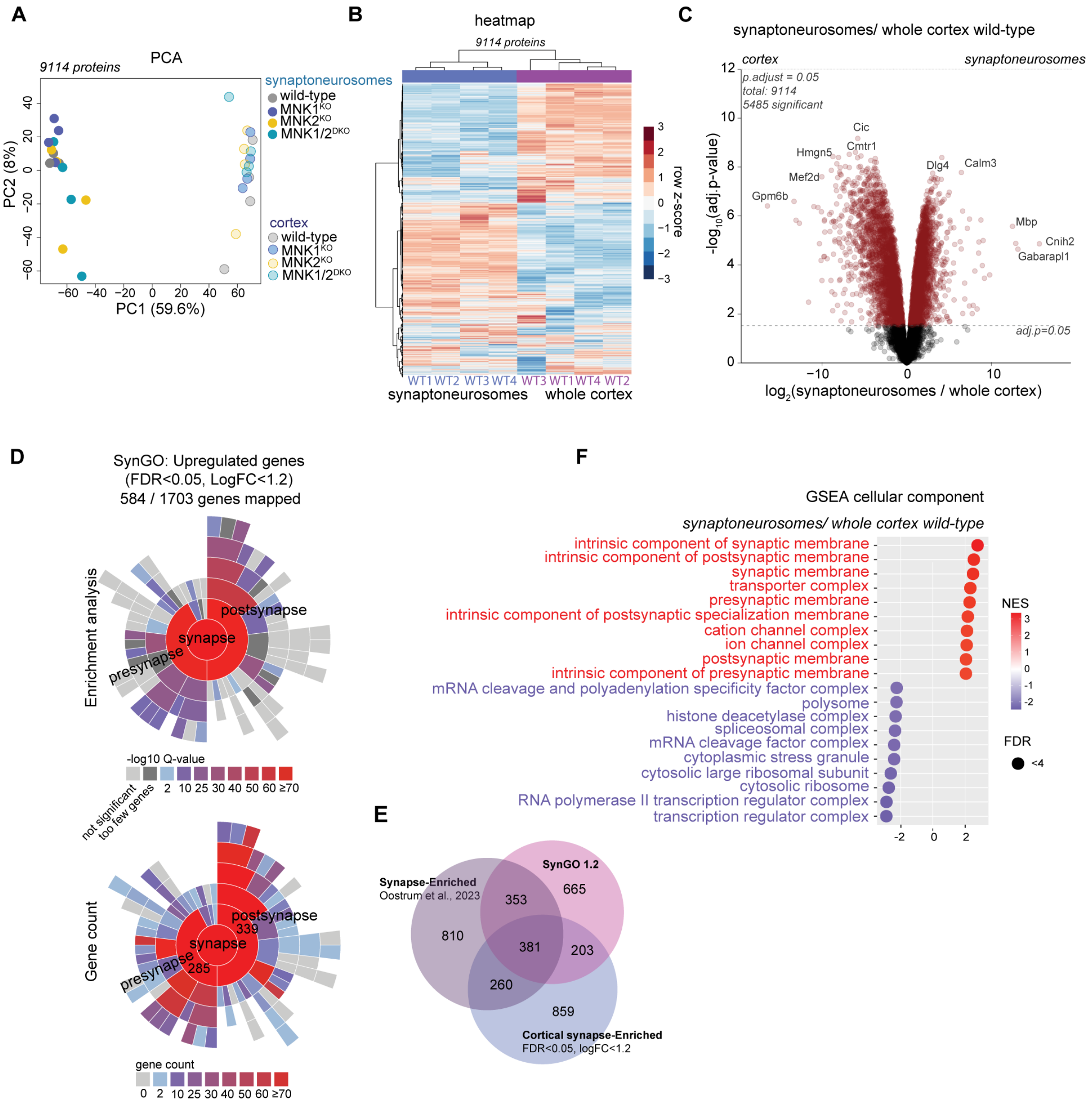

Supplementary Figure 4. Validation of the synaptoneurosome enrichment.
(A) PCA showing a clear separation between the proteomes from synaptoneurosomes and cortex in all genotypes. (B) Hierarchical clustering of normalized protein expression of all proteins detected in synaptoneurosomes and corex from wild-type mice. (C) Volcano plot comparing wild-type synaptoneurosomes to cortex. Differentially expressed proteins (P.adjust<0.05) are marked in red. Over half of all identified proteins were significantly altered in the isolated synaptoneurosome fractions. (D) SynGO (Koopmans et al., 2019) sunburst plot showing enrichment analysis (top) and gene count (bottom) of upregulated proteins (FDR<0.05, logFC<1.2). We found a significant overrepresentation of pre-and postsynaptic proteins in the synaptoneurosome fraction, with a similar number of pre-and postsynaptic proteins identified. (E) Venn diagram of the overlap between cortical synapse-enriched proteins to proteins identified as synapse-enriched in the SynGO database or the synapse-enriched proteins from Oostrum et al., (2023). We found a good representation of known synaptic proteins enriched in the synaptoneurosome fraction, with around 50% of the enriched proteins present in either dataset. (F) Gene set enrichment analysis (GSEA) of cellular components for wild-type synaptoneurosomes compared to cortex showed enrichment of genes linked to the synapse and decrease of proteins associated with the nucleus and cytosol. Top ten increased (red) and decreased (blue) pathways are shown. NES= normalized enrichment score.

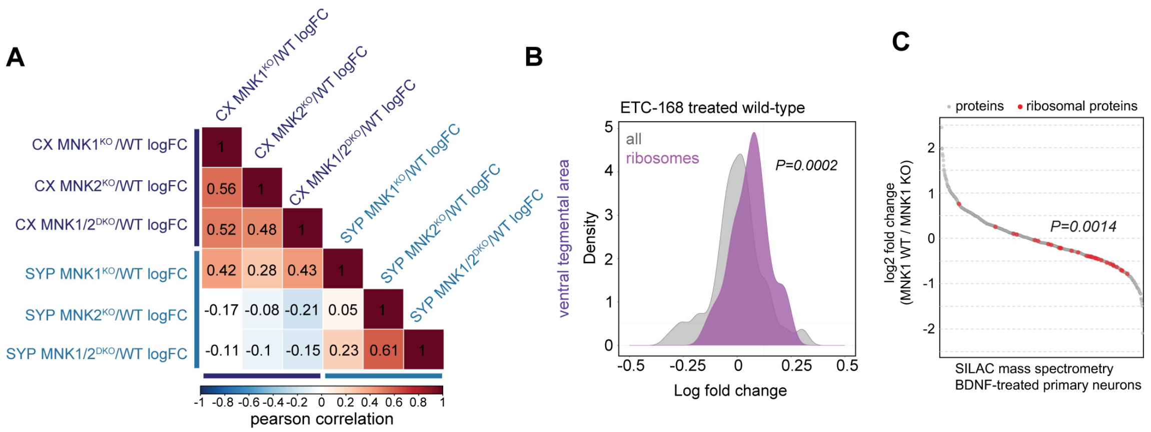

Supplementary Figure 5. Comparison between the cortical and synaptic proteome shows site-specific effects of MNK2 deletion.

(A) Correlation matrix heatmap showing the Pearson correlation of logFC values of the proteome from cortex and synaptoneurosomes in MNK1^{KO}, MNK2^{KO}, and MNK1/2^{DKO} relative to wild-type. (B) Density plots of logFC ribosomal protein abundance compared to all proteins from wild-type mice treated with ETC-168 compared to vehicle. The proteomic data is from Hörnberg et al., 2020. (C) SILAC proteomic from BDNF-treated MNK1^{WT} and MNK1^{KO} primary neurons. The plot shows the heavy medium (H/M) MNK1WT/KO ratio. Ribosomal proteins are marked in red. Data from Genheden et al., 2015. P-value was calculated using a two-sided Kolmogorov-Smirnov test for B, and Wilcoxon-Mann-Whitney-U test for C.

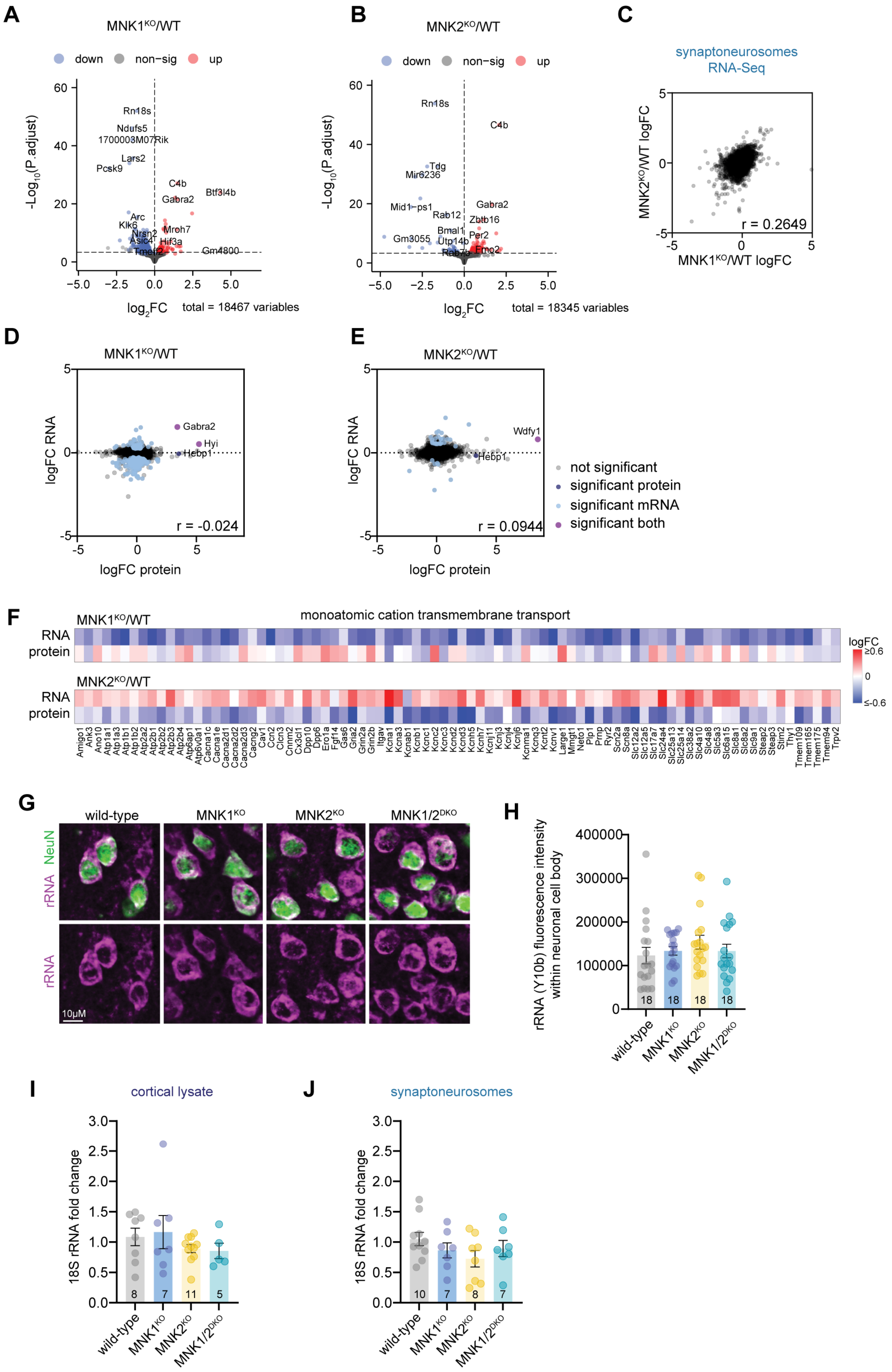
A
MNK1KO/WT
down non−sig up
-Log10(P.adjust)
log2FC
total = 18467 variables
Rn18s
Ndufs5
1700003M07Rik
Lars2
Pcsk9
C4b
Btf3l4b
Gabra2
Arc
Klk6
Nrsn2
Asic4
Tmeff2
Mroh7
Hif3a
Gm4800
B
MNK2KO/WT
down non−sig up
-Log10(P.adjust)
log2FC
total = 18345 variables
Rn18s
C4b
Tdg
Mir6236
Mid1−ps1
Rab12
Gabra2
Zbtb16
Bmal1
Per2
Gm3055
Utp14b
Rab7b
Fmo2
C
synaptoneurosomes
RNA-Seq
MNK2KO/WT logFC
MNK1KO/WT logFC
r = 0.2649
D
MNK1KO/WT
logFC RNA
logFC protein
Gabra2
Hyi
Hebp1
r = -0.024
E
MNK2KO/WT
logFC RNA
logFC protein
Wdfy1
Hebp1
r = 0.0944
not significant
significant protein
significant mRNA
significant both
F
MNK1KO/WT
monoatomic cation transmembrane transport
RNA
protein
MNK2KO/WT
RNA
protein
logFC
≥0.6
0
≤-0.6
G
wild-type
MNK1KO
MNK2KO
MNK1/2DKO
NeuN
rRNA
rRNA
10μM
H
rRNA (Y10b) fluorescence intensity within neuronal cell body
wild-type
MNK1KO
MNK2KO
MNK1/2DKO
I
cortical lysate
18S rRNA fold change
wild-type
MNK1KO
MNK2KO
MNK1/2DKO
J
synaptoneurosomes
18S rRNA fold change
wild-type
MNK1KO
MNK2KO
MNK1/2DKO

Supplementary Figure 6. Knockout of MNK1 or MNK2 has different effects on the synaptic transcriptome.

(A-B) Volcano plot of mRNA in synaptoneurosomes from (A) $MNK1^{KO}$ and (B) $MNK2^{KO}$ mice. Downregulated genes (P.adjust<0.05) are shown in blue, and upregulated in red. (C) Alterations in mRNA from $MNK1^{KO}$ synaptoneurosomes compared to $MNK2^{KO}$ synaptoneurosomes relative to WT. r was determined by Pearson correlation. (D-E) Comparison of mRNA and protein expression in $MNK1^{KO}$ synaptoneurosomes relative to WT (D) and $MNK2^{KO}$ synaptoneurosomes relative to WT (E). Only genes detected on both the RNA and protein levels in all genotypes were plotted. r was determined by Pearson correlation. (F) Heatmap showing logFC of core enriched proteins and mRNAs in the GO term Monoatomic cation transmembrane transport. Both the mRNA and protein are from isolated synaptoneurosomes from $MNK1^{KO}$ mice (top) and $MNK2^{KO}$ mice (bottom), relative to wild-type mice. (G-H) Representative image (G) and quantification (H) of rRNA labeled with the Y10b antibody in cortex from wild-type, $MNK1^{KO}$, $MNK2^{KO}$, and $MNK1/2^{DKO}$. N: mean fluorescent intensity of neurons from 18 images from 3 animals per genotype, 5-7 images per animal. (I-J) Relative fold change of 18S rRNA to the mean of wild-type control in cortex (I) and synaptoneurosomes (J). The samples were normalized to GAPDH. Error bars show s.e.m. Significance was determined by Kruskal-Wallis test for H, I, and J.

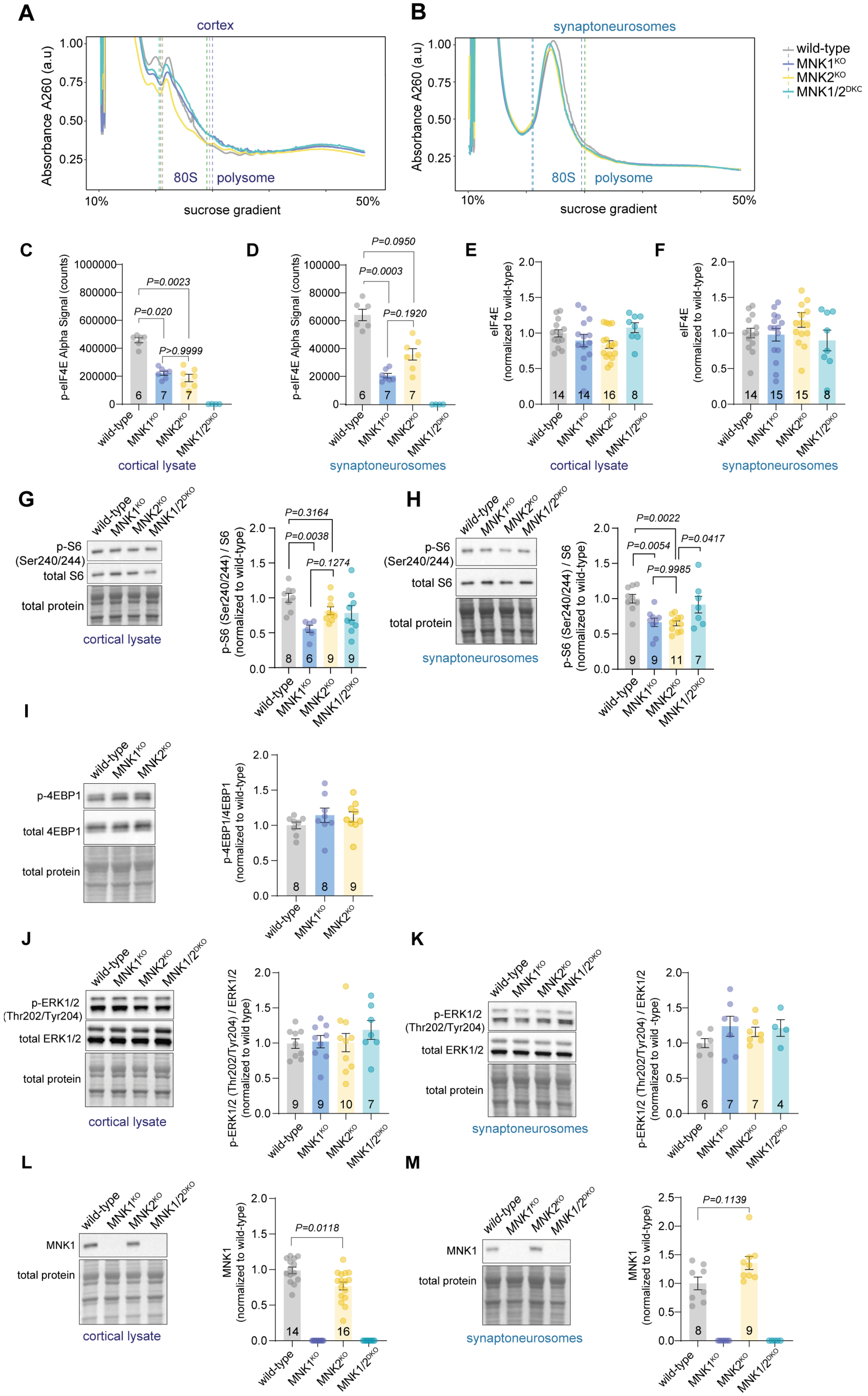
A
cortex
Absorbance A260 (a.u)
80S
polysome
10%
sucrose gradient
50%
B
synaptoneurosomes
wild-type
MNK1KO
MNK2KO
MNK1/2DKC
C
p-eIF4E Alpha Signal (counts)
P=0.0023
P=0.020
P>0.9999
cortical lysate
D
P=0.0950
P=0.0003
P=0.1920
synaptoneurosomes
E
eIF4E (normalized to wild-type)
cortical lysate
F
synaptoneurosomes
G
p-S6 (Ser240/244)
total S6
total protein
cortical lysate
P=0.3164
P=0.0038
P=0.1274
p-S6 (Ser240/244) / S6 (normalized to wild-type)
H
synaptoneurosomes
P=0.0022
P=0.0054
P=0.0417
P=0.9985
I
p-4EBP1
total 4EBP1
total protein
p-4EBP1/4EBP1 (normalized to wild-type)
J
p-ERK1/2 (Thr202/Tyr204)
total ERK1/2
total protein
cortical lysate
p-ERK1/2 (Thr202/Tyr204) / ERK1/2 (normalized to wild type)
K
synaptoneurosomes
L
MNK1
total protein
cortical lysate
P=0.0118
MNK1 (normalized to wild-type)
M
synaptoneurosomes
P=0.1139
wild-type
MNK1KO
MNK2KO
MNK1/2DKO

Supplementary Figure 7. Effect of MNK1 and MNK2 deletion on protein abundance and phosphorylation levels in pathways related to protein synthesis.

(A-B) Representative traces from polysome profiling of (A) cortex and (B) synaptoneurosomes. The lines represent the start and end of the monosome (80S) fraction. (C-D) Normalized p-eIF4E AlphaLisa count from (C) cortical lysate and (D) synaptoneurosomes from wild-type, $MNK1^{KO}$, and $MNK2^{KO}$ mice. $MNK1/2^{DKO}$ are included as validation. (E-F) Quantification of eIF4E from wild-type, $MNK1^{KO}$, $MNK2^{KO}$, and $MNK1/2^{DKO}$ mice in (E) cortical lysate and (F) synaptoneurosomes. Images are in Figure 6K-L. (G-H) Representative western blot (left) and quantification (right) of p-rpS6 compared to total rpS6 from (G) cortical lysates and (H) synaptoneurosomes from wild-type, $MNK1^{KO}$, $MNK2^{KO}$, and $MNK1/2^{DKO}$ mice. (I) Representative image and quantification of p-4EBP1 compared to total 4EBP1 in synaptoneurosomes from wild-type, $MNK1^{KO}$, and $MNK2^{KO}$ mice. (J-K) Representative image and quantification p-ERK1/2 compared to total ERK1/2 in wild-type, $MNK1^{KO}$, $MNK2^{KO}$, and $MNK1/2^{DKO}$ mice in (J) cortical lysate and (K) synaptoneurosomes. (L-M) Representative image and quantification of MNK1 in wild-type and $MNK2^{KO}$ mice in (L) cortical lysate and (M) synaptoneurosomes. $MNK1^{KO}$ and $MNK1/2^{DKO}$ mice are included as validation. Significance was determined by Kruskal-Wallis test followed by Dunn's multiple comparison test for C-D, one-way ANOVA followed by Tukey's multiple comparison test for E-K, and Mann-Whitney test for L-M.

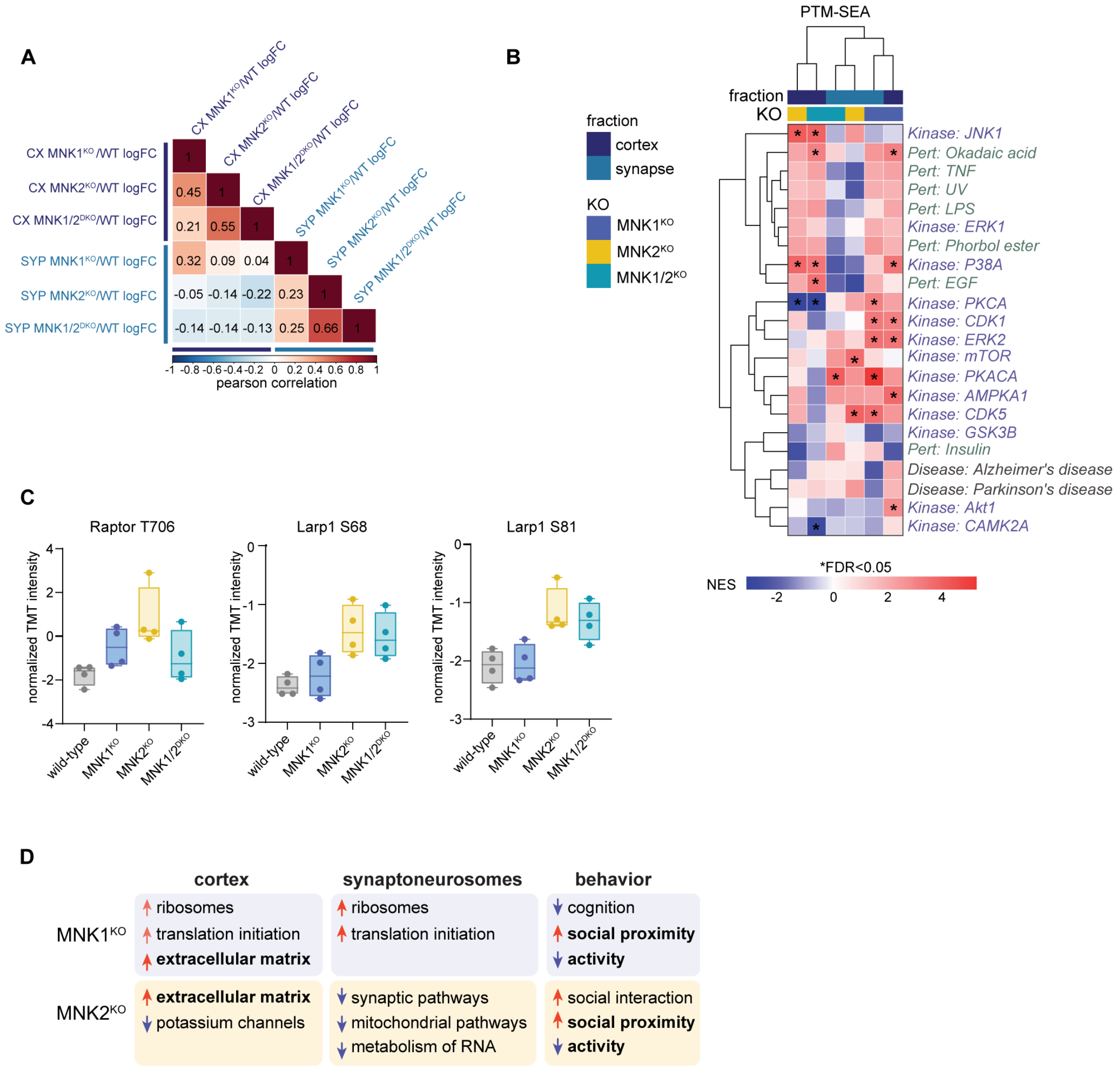

Supplementary Figure 8. Effect of MNK1 and MNK2 deletion on the cortical and synaptic phosphoproteome.

(A) Correlation matrix heatmap showing Pearson correlation of cortical and synaptoneurosomes phosphoproteome logFC values in MNK1^{KO}, MNK2^{KO}, and MNK1/2^{DKO} relative to wild-type. (B) Heatmap showing hierarchical clustering of phosphosite-specific signature normalized enrichment scores (NES) as determined by PTM-SEA. Significant changes (P.adjust<0.05) are marked with *. (C) Normalized TMT intensities for phosphosites linked to the mTOR pathway increased in synaptoneurosomes from MNK2^{KO} mice compared to MNK1^{KO} mice. Error bars in C show min to max. (D) Graphical representation of the molecular and behavioral phenotypes in MNK1^{KO} and MNK2^{KO} mice. Terms that overlap between the genotypes are marked in bold.
