# Supplementary material for: Distinct roles for MNK1 and MNK2 in social and cognitive behavior through kinase-specific regulation of the synaptic proteome and phosphoproteome
Source: Mol Psychiatry. 2026 Feb 13;31(6):3446–61. doi: 10.1038/s41380-026-03483-w (PMC13190234; doi:10.1038/s41380-026-03483-w)
Supplement: Supplementary file 2 — Supplementary Table 1 [file 41380_2026_3483_MOESM2_ESM.pdf]

| Test | Sample Size | Statistical test | Values |
|---|---|---|---|
| **wild-type, MNK1$^{KO}$, MNK2$^{KO}$** | | | |
| Social Habituation test | *females:*<br>wild-type=10<br>MNK1$^{KO}$=8<br>MNK2$^{KO}$=12<br><br>*males:*<br>wild-type=7<br>MNK1$^{KO}$=6<br>MNK2$^{KO}$=5 | Three-way Repeated Measures ANOVA | Genotype: $F_{(2, 42)}$=0.28, P=0.760<br>Sex: $F_{(1, 42)}$=1.19, P=0.2820<br>genotype x Sex $F_{(2, 42)}$=1.63, P=0.2080<br>trials: $F_{(3.35, 140.79)}$=81.16, P<0.0001<br>genotype x trials: $F_{(6.7, 140.79)}$=1.887, P=0.0820<br>Sex x trials $_{(3.35, 140.79)}$: F=0.86, P=0.4720<br>Sex x trials x genotype $_{(6.7, 140.79)}$: F=0.67, P=0.6880 |
| Social recognition index | *females:*<br>wild-type=10<br>MNK1$^{KO}$=8<br>MNK2$^{KO}$=12<br><br>*males:*<br>wild-type=7<br>MNK1$^{KO}$=6<br>MNK2$^{KO}$=5 | Two-way ANOVA, Tukey's post-hoc test for multiple comparisons | Interaction: F $_{(2, 42)}$ = 0.2222, P=0.8017<br>Sex: F $_{(1, 42)}$ = 0.2822, P=0.5980<br>Genotype: F $_{(2, 42)}$ = 6.141, P=0.0046<br><br>wild-type vs MNK1$^{KO}$: P= 0.0060<br>wild-type vs MNK2$^{KO}$: P= 0.9260<br>MNK1$^{KO}$ vs MNK2$^{KO}$: P = 0.0209 |
| Social olfaction | *females:*<br>wild-type=10<br>MNK1$^{KO}$=8<br>MNK2$^{KO}$=12<br><br>*males:*<br>wild-type=7<br>MNK1$^{KO}$=7<br>MNK2$^{KO}$=5 | Three-way Repeated Measures ANOVA | Genotype: $F_{(2, 43)}$ =2.48, P=0.0950<br>Sex: $F_{(1, 43)}$ =2.23, P=0.1430<br>genotype x sex: $F_{(2, 42)}$ =2.06, P=0.139<br>trials: $F_{(3.87, 166.45)}$ =42.84, P<0.0001<br>genotype x trials: $F_{(7.74, 166.45)}$ =1.47, P=0.175<br>Sex x trials: $F_{(3.87, 166.45)}$ =0.58, P=0.669<br>Sex x trials x genotype: $F_{(7.74, 166.45)}$ =1.62, P=0.125 |
| Object habituation test | *females:*<br>wild-type=10<br>MNK1$^{KO}$=8<br>MNK2$^{KO}$=12<br><br>*males:*<br>wild-type=7<br>MNK1$^{KO}$=7<br>MNK2$^{KO}$=5 | Three-way Repeated Measures ANOVA | Genotype: $F_{(2, 43)}$ =9.80, P<0.0001<br>Sex: $F_{(1, 43)}$ =0.54, P=0.465<br>genotype x sex: $F_{(2, 43)}$ =1.74, P=0.188<br>trials: $F_{(2.43, 104.6)}$ =34.96, P<0.0001<br>genotype x trials: $F_{(4.86, 104.6)}$ =5.89, P<0.0001<br>Sex x trials: $F_{(2.43, 104.6)}$ =0.83, P=0.461<br>Sex x trials x genotype: $F_{(4.86, 104.6)}$ =0.61, P=0.689 |
| Object RI | *females:*<br>wild-type=10<br>MNK1$^{KO}$=8<br>MNK2$^{KO}$=12 | Two-way ANOVA, Tukey's post-hoc test for multiple comparisons | Interaction: F $_{(2, 43)}$ = 0.9540, P=0.3932<br>Sex: F $_{(1, 43)}$ = 0.6882, P=0.4114<br>Genotype: F $_{(2, 43)}$ = 14.51, P<0.0001 |

| | | | |
|---|---|---|---|
| | *males:*<br>wild-type=7<br>MNK1$^{KO}$=7<br>MNK2$^{KO}$=5 | | Wild-type vs MNK1$^{KO}$ : P=0.0221<br>Wild-type vs MNK2$^{KO}$: P=0.0211<br>MNK1$^{KO}$ vs MNK2$^{KO}$: P<0.0001 |
| NOR | *females:*<br>wild-type=10<br>MNK1$^{KO}$=8<br>MNK2$^{KO}$=12<br><br>*males:*<br>wild-type=7<br>MNK1$^{KO}$=7<br>MNK2$^{KO}$=5 | Three-way Repeated Measures ANOVA, Bonferroni's post-hoc test for multiple comparisons | Genotype: $F_{(2, 43)}$ =11.66, P<0.0001<br>Sex: $F_{(1, 43)}$ =0.51, P=0.479<br>Novelty: $F_{(1, 43)}$ =21.05, P<0.0001<br>genotype x sex: $F_{(2, 43)}$ =3.23, P=0.049<br>genotype x novelty: $F_{(2, 43)}$ =4.62, P=0.015<br>Sex x novelty: $F_{(1, 43)}$ =0.70, P=0.409<br>Sex x novelty x genotype: $F_{(2, 43)}$ =0.52, P=0.596<br><br><u>Post-hoc: Novelty x genotype</u><br>*Familiar vs novel object*<br>Wild-type: P=0.004<br>MNK1$^{KO}$: P=0.772<br>MNK2$^{KO}$: P=0.003<br><br><u>Post-hoc: Novelty x sex</u><br>*female*<br>Wild-type vs MNK1$^{KO}$ : P=0.0056<br>Wild-type vs MNK2$^{KO}$: P=0.056<br>MNK1$^{KO}$ vs MNK2$^{KO}$: P<0.0001<br><br>*male*<br>Wild-type vs MNK1$^{KO}$: P=0.158<br>Wild-type vs MNK2$^{KO}$: P>0.9999<br>MNK1$^{KO}$ vs MNK2$^{KO}$: P=0.687<br><br><u>Post-hoc: Novelty x sex</u><br>*Female vs male*<br>Wild-type: P=0.38<br>MNK1$^{KO}$: P=0.017<br>MNK2$^{KO}$: P=0.115 |
| NOR RI | females:<br>wild-type=10<br>MNK1$^{KO}$=8<br>MNK2$^{KO}$=12<br><br>males:<br>wild-type=7<br>MNK1$^{KO}$=7<br>MNK2$^{KO}$=5 | Two-way ANOVA, Tukey's post-hoc test for multiple comparisons | Interaction: $F_{(2, 43)}$ = 0.8828, P=0.4210<br>Sex: $F_{(1, 43)}$ = 1.423, P=0.2394<br>Genotype: $F_{(2, 43)}$ = 5.574, P=0.0070<br><br>Wild-type vs MNK1$^{KO}$ : P=0.0497<br>Wild-type vs MNK2$^{KO}$: P=0.6531<br>MNK1$^{KO}$ vs MNK2$^{KO}$: P=0.0071 |
| Open field distance traveled | females:<br>wild-type=10<br>MNK1$^{KO}$=6<br>MNK2$^{KO}$=12<br><br>males:<br>wild-type=6<br>MNK1$^{KO}$=7<br>MNK2$^{KO}$=5 | Two-way ANOVA | Interaction: $F_{(2, 40)}$ = 2.049, P=0.1422<br>Sex: $F_{(1, 40)}$ = 0.01534, P=0.9020<br>Genotype: $F_{(2, 40)}$ = 2.453, P=0.0989 |

| | | | |
|---|---|---|---|
| Time in center | females:<br>wild-type=10<br>$MNK1^{KO}$=6<br>$MNK2^{KO}$=12<br><br>males:<br>wild-type=6<br>$MNK1^{KO}$=7<br>$MNK2^{KO}$=5 | Two-way ANOVA | Interaction: $F_{(2, 40)} = 0.1024$, P=0.9029<br>Sex: $F_{(1, 40)} = 2.415$, P=0.1281<br>Genotype: $F_{(2, 40)} = 0.1471$, P=0.8637 |
| wild type, $MNK1/2^{DKO}$ | | | |
| Social Habituation test | females:<br>wild-type=8<br>$MNK1/2^{DKO}$ =8<br><br>males:<br>wild-type=5<br>$MNK1/2^{DKO}$ =7 | Three-way Repeated Measures ANOVA | Genotype: $F_{(1, 24)}$ =0.18, P=0.671<br>Sex: $F_{(1, 24)}$ =3.51, P=0.073<br>genotype x sex: $F_{(1, 24)}$ =0.27, P=0.607<br>trials: $F_{(2.6, 62.31)}$ =30.47, P<0.0001<br>genotype x trials: $F_{(2.6, 62.31)}$ =0.3, P=0.794<br>Sex x trials: $F_{(2.6, 62.31)}$ =0.74, P=0.516<br>Sex x trials x genotype $F_{(2.6, 62.31)}$ =2.57, P=0.07 |
| Social recognition index | females:<br>wild-type=8<br>$MNK1/2^{DKO}$ =8<br><br>males:<br>wild-type=5<br>$MNK1/2^{DKO}$ =7 | Two-way ANOVA | Interaction: $F_{(1, 24)} = 0.06461$, P=0.8015<br>Sex: $F_{(1, 24)} = 1.246$, P=0.2753<br>Genotype: $F_{(1, 24)} = 0.001524$, P=0.9692 |
| Social olfaction | females:<br>wild-type=8<br>$MNK1/2^{DKO}$ =8<br><br>males:<br>wild-type=5<br>$MNK1/2^{DKO}$ =8 | Three-way Repeated Measures ANOVA | Genotype: $F_{(1, 25)}$ =0.83, P=0.371<br>Sex: $F_{(1, 25)}$ =0.9, P=0.351<br>genotype x sex: $F_{(1, 25)}$ =3.51, P=0.073<br>trials: $F_{(3.96, 98.97)}$ =18.17, P<0.0001<br>genotype x trials: $F_{(3.96, 98.97)}$ =0.38, P=0.819<br>Sex x trials: $F_{(3.96, 98.97)}$ =0.42, P=0.791<br>Sex x trials x genotype $F_{(3.96, 98.97)}$ =0.69, P=0.602 |
| Object Habituation test | females:<br>wild-type=8<br>$MNK1/2^{DKO}$ =8<br><br>males:<br>wild-type=5<br>$MNK1/2^{DKO}$ =8 | Three-way Repeated Measures ANOVA, Bonferroni's post-hoc test for multiple comparisons | Genotype: $F_{(1, 25)}$ =0.69, P=0.415<br>Sex: $F_{(1, 25)}$ =4.49, P=0.044<br>genotype x sex: $F_{(1, 25)}$ =0.85, P=0.364<br>trials: $F_{(2.42, 60.55)}$ =24.34, P<0.0001<br>genotype x trials: $F_{(2.42, 60.55)}$ =0.49, P=0.652<br>Sex x trials: $F_{(2.42, 60.55)}$ =1.27, P=0.292<br>Sex x trials x genotype $F_{(2.42, 60.55)}$ =4.29, P=0.013 |

| | | | |
|---|---|---|---|
| | | | <u>Trial 1:</u><br>WT f vs DKO f: P=0.136<br>DKO f vs WT m:P=0.157<br>WT f vs DKO m:P=1<br>DKO f vs WT m:P=1<br>WT f vs WT m:P=0.423<br>DKO m vs WT m:P=0.475<br><br><u>Trial 2:</u><br>WT f vs DKO f: P=1<br>DKO f vs WT m:P=0.167<br>WT f vs DKO m:P=1<br>DKO f vs WT m:P=1<br>WT f vs WT m:P=1<br>DKO m vs WT m:P=0.786<br><br><u>Trial 3:</u><br>WT f vs DKO f: P=1<br>DKO f vs WT m:P=0.298<br>WT f vs DKO m:P=0.16<br>DKO f vs WT m:P=1<br>WT f vs WT m:P=1<br>DKO m vs WT m:P=1<br><br><u>Trial 4:</u><br>WT f vs DKO f: P=0.95<br>DKO f vs WT m:P=0.648<br>WT f vs DKO m:P=0.027<br>DKO f vs WT m:P=1<br>WT f vs WT m:P=0.388<br>DKO m vs WT m:P=1<br><br><u>Trial 5:</u><br>WT f vs DKO f: P=1<br>DKO f vs WT m:P=1<br>WT f vs DKO m:P=0.508<br>DKO f vs WT m:P=1<br>WT f vs WT m:P=0.579<br>DKO m vs WT m:P=1 |
| Object recognition index | females:<br>wild-type=8<br>MNK1/2$^{DKO}$ =8<br><br>males:<br>wild-type=5<br>MNK1/2$^{DKO}$ =8 | Two-way ANOVA | Interaction: $F_{(1, 25)}$ = 0.7184, P=0.4047<br>Sex: $F_{(1, 25)}$ = 0.08981, P=0.7669<br>Genotype: $F_{(1, 25)}$ = 0.04815, P=0.8281 |
| NOR | MNK1/2$^{DKO}$ male=8<br>MNK1/2$^{DKO}$ female=8 | Three-way Repeated Measures ANOVA | Genotype: $F_{(1, 25)}$ =0.08, P=0.78<br>Sex: $F_{(1, 25)}$ =1.82, P=0.19<br>Novelty: $F_{(1, 25)}$ =27.51, P<0.0001<br>genotype x sex: $F_{(1, 25)}$ =0.06, P=0.802<br>genotype x novelty: $F_{(1, 25)}$ =0.6, P=0.446<br>Sex x novelty: $F_{(1, 25)}$ =4.79, P=0.038<br>Sex x novelty x genotype: $F_{(1, 25)}$ =0.14, P=0.712 |

| | | | |
|---|---|---|---|
| | | | Post-hoc: Novelty x sex<br>Familiar vs novel females : P<0.0001<br>Familiar vs novel males: P=0.072 |
| NOR RI | females:<br>wild-type=8<br>MNK1/2$^{DKO}$ =8<br><br>males:<br>wild-type=5<br>MNK1/2$^{DKO}$ =8 | Two-way ANOVA | Interaction: $F_{(1, 25)}$ = 0.06243, P=0.8047<br>Sex: $F_{(1, 25)}$ = 02.876, P=5188<br>Genotype: $F_{(1, 25)}$ = 0.4284, P=0.1023 |
| Open field | females:<br>wild-type=8<br>MNK1/2$^{DKO}$ =8<br><br>males:<br>wild-type=5<br>MNK1/2$^{DKO}$ =8 | Two-way ANOVA, Tukey’s post-hoc test for multiple comparisons | Interaction: $F_{(1, 25)}$ = 0.1033, P=0.7505<br>Sex: $F_{(1, 25)}$ = 0.9874, P=0.3299<br>Genotype: $F_{(1, 25)}$ = 15.19, P=0.0005<br><br>Wild-type females vs MNK1/2$^{DKO}$ females: P=0.0155<br>Wild-type males vs MNK1/2$^{DKO}$ males: P=0.0043 |
| Time in center | females:<br>wild-type=8<br>MNK1/2$^{DKO}$ =8<br><br>males:<br>wild-type=5<br>MNK1/2$^{DKO}$ =8 | Two-way ANOVA | Interaction: $F_{(1, 25)}$ = 0.8855, P=0.3557<br>Sex: $F_{(1, 25)}$ = 0.1842, P=0.6715<br>Genotype: $F_{(1, 25)}$ = 0.2072, P=0.6529 |
