# Supplementary material for: Distinct roles for MNK1 and MNK2 in social and cognitive behavior through kinase-specific regulation of the synaptic proteome and phosphoproteome
Source: Mol Psychiatry. 2026 Feb 13;31(6):3446–61. doi: 10.1038/s41380-026-03483-w (PMC13190234; doi:10.1038/s41380-026-03483-w)
Supplement: Supplementary file 8 — Supplementary Table 7 [file 41380_2026_3483_MOESM8_ESM.pdf]

Figure	Sample Size	Statistical test	Values
1B	wild-type=17<br>MNK1^{KO}=14<br>MNK2^{KO}=17<br><br>*Trial 5 is missing from one MNK2^{KO} mouse*	Mixed-effects model, Tukey's post-hoc test for multiple comparisons	trial: $F_{(3.512, 157.2)} = 8.760$, P<0.0001<br>genotype: $F_{(2, 45)} = 4.735$, P=0.0136<br>trial × genotype interaction: $F_{(8, 179)} = 0.8201$, P=0.5858<br><br>wild-type vs MNK1^{KO}<br>Trial1: P=0.1996<br>Trial2: P=0.4601<br>Trial3: P=0.8604<br>Trial4: P=0.1793<br>Trial5: P=0.2983<br><br>wild-type vs MNK2^{KO}<br>Trial1: P=0.0500<br>Trial2: P=0.1476<br>Trial3: P=0.2713<br>Trial4: P=0.0609<br>Trial5: P=0.2653<br><br>MNK1^{KO} vs MNK2^{KO}<br>Trial1: P=0.9361<br>Trial2: P=0.7916<br>Trial3: P=0.0967<br>Trial4: P=0.9844<br>Trial5: P=0.9880
1C	wild-type=17<br>MNK1^{KO}=14<br>MNK2^{KO}=17<br><br>*Trial 5 is missing from one MNK2^{KO} mouse*	Mixed-effects model, Tukey's post-hoc test for multiple comparisons	trial: $F_{(3.318, 148.5)} = 0.5512$, P=0.6658<br>genotype: $F_{(2, 45)} = 6.569$, P=0.0031<br>trial × genotype interaction: $F_{(8, 179)} = 0.9465$, P=0.4797<br><br>wild-type vs MNK1^{KO}<br>Trial1: P=0.0226<br>Trial2: P=0.0063<br>Trial3: P=0.5086<br>Trial4: P=0.7346<br>Trial5: P=0.066<br><br>wild-type vs MNK2^{KO}<br>Trial1: P=0.2453<br>Trial2: P=0.1319<br>Trial3: P=0.2567<br>Trial4: P=0.1472<br>Trial5: P=0.598<br><br>MNK1^{KO} vs MNK2^{KO}<br>Trial1: P=0.6477<br>Trial2: P=0.3311<br>Trial3: P=0.8572<br>Trial4: P=0.5895

			Trial5: P=0.4593
1D	wild-type=17<br>$MNK1^{KO}$=14<br>$MNK2^{KO}$=17<br><br>*Trial 5 is missing from one $MNK2^{KO}$ mouse*	Mixed-effects model, Tukey's post-hoc test for multiple comparisons	trial: $F_{(2.703, 121.0)} = 3.131$, P=0.0328<br>genotype: $F_{(2, 45)} = 13.24$, P<0.0001<br>trial × genotype interaction: $F_{(8, 179)} = 1.357$, P=0.2183<br><br>wild-type vs $MNK1^{KO}$<br>Trial1: P=0.357<br>Trial2: P=0.0155<br>Trial3: P=0.2736<br>Trial4: P=0.0188<br>Trial5: P=0.0886<br><br>wild-type vs $MNK2^{KO}$<br>Trial1: P=0.0004<br>Trial2: P=0.0001<br>Trial3: P=0.0351<br>Trial4: P=0.3417<br>Trial5: P=0.0667<br><br>$MNK1^{KO}$ vs $MNK2^{KO}$<br>Trial1: P=0.2773<br>Trial2: P=0.6374<br>Trial3: P=0.4472<br>Trial4: P=0.2077<br>Trial5: P=0.9735
1E	wild-type=17<br>$MNK1^{KO}$=15<br>$MNK2^{KO}$=17	RM two-way ANOVA	trials: $F_{(3.882, 178.6)} = 41.79$, P<0.0001<br>genotype: $F_{(2, 46)} = 1.825$, P=0.1727<br>trials × genotype: $F_{(22, 506)} = 1.265$, P=0.1887
1F	wild-type=17<br>$MNK1^{KO}$=15<br>$MNK2^{KO}$=17	RM two-way ANOVA, Tukey's post-hoc test for multiple comparisons	trial: $F_{(2.514, 115.6)} = 37.21$, P<0.0001<br>genotype: $F_{(2, 46)} = 10.58$, P=0.0002<br>trial × genotype: $F_{(8, 184)} = 6.180$, P<0.0001<br><br>wild-type vs $MNK1^{KO}$<br>Trial1: P=0.0037<br>Trial2: P=0.0155<br>Trial3: P=0.1663<br>Trial4: P=0.0127<br>Trial5: P=0.0005<br><br>wild-type vs $MNK2^{KO}$<br>Trial1: P=0.8023<br>Trial2: P=0.8813<br>Trial3: P=0.7782

			Trial4: P=0.9338<br>Trial5: P=0.2891<br><br>$MNK1^{KO}$ vs $MNK2^{KO}$<br>Trial1: P=0.0066<br>Trial2: P=0.0309<br>Trial3: P=0.3216<br>Trial4: P=0.0086<br>Trial5: P<0.0001
1G	wild-type=17<br>$MNK1^{KO}$=15<br>$MNK2^{KO}$=17	Kruskal-Wallis test, Dunn's post-hoc test for multiple comparisons	$K_{(3)}$=19.25, P<0.0001<br><br>wild-type vs $MNK1^{KO}$: P=0.0073<br>wild-type vs $MNK2^{KO}$: P=0.5720<br>$MNK1^{KO}$ vs $MNK2^{KO}$: P<0.0001
1H	wild-type=17<br>$MNK1^{KO}$=15<br>$MNK2^{KO}$=17	RM two-way ANOVA, Tukey's post-hoc test for multiple comparisons	object: $F_{(1, 46)}$ = 20.08, P<0.0001<br>genotype: $F_{(2, 46)}$ = 13.48, P<0.0001<br>object $\times$ genotype: $F_{(2, 46)}$ = 4.183, P=0.0214<br><br>familiar vs novel<br>wild-type: P=0.0006<br>$MNK1^{KO}$: P=0.9973<br>$MNK2^{KO}$: P=0.0017
1I	wild-type=17<br>$MNK1^{KO}$=15<br>$MNK2^{KO}$=17	One-way ANOVA, Tukey's post-hoc test for multiple comparisons	$F_{(2, 46)}$ = 4.843, P=0.0123<br><br>wild-type vs $MNK1^{KO}$: P= 0.0407<br>wild-type vs $MNK2^{KO}$: P= 0.9177<br>$MNK1^{KO}$ vs $MNK2^{KO}$: P= 0.0158
1J	wild-type=16<br>$MNK1^{KO}$=15<br>$MNK2^{KO}$=17<br><br>*Last three minutes are missing from one $MNK1^{KO}$ mouse*	Mixed-effects model, Tukey's post-hoc test for multiple comparisons	time: $F_{(4.129, 183.0)}$ = 20.74, P<0.0001<br>genotype: $F_{(2, 45)}$ = 3.984, P=0.0255<br>time $\times$ genotype: $F_{(18, 399)}$ = 3.815, P<0.0001<br><br>wild-type vs $MNK1^{KO}$<br>Minute 1: P=0.0640<br>Minute 2: P=0.0006<br>Minute 3: P=0.0652<br>Minute 4: P=0.1525<br>Minute 5: P=0.0203<br>Minute 6: P=0.3122<br>Minute 7: P=0.7071<br>Minute 8: P=0.8076<br>Minute 9: P=0.6218<br>Minute 10: P=0.2908<br><br>wild-type vs $MNK2^{KO}$<br>Minute 1: P=0.1390<br>Minute 2: P=0.4599

			Minute 3: P=0.2032<br>Minute 4: P=0.4710<br>Minute 5: P=0.0012<br>Minute 6: P=0.1773<br>Minute 7: P=0.9988<br>Minute 8: P=0.8445<br>Minute 9: P=0.6583<br>Minute 10: P=0.2778<br><br>$MNK1^{KO}$ vs $MNK2^{KO}$<br>Minute 1: P=0.0003<br>Minute 2: P=0.0266<br>Minute 3: P=0.6507<br>Minute 4: P=0.5080<br>Minute 5: P=0.6591<br>Minute 6: P=0.8818<br>Minute 7: P=0.6743<br>Minute 8: P=0.3860<br>Minute 9: P=0.9824<br>Minute 10: P=0.9967
1K	wild-type=16<br>$MNK1^{KO}$=15<br>$MNK2^{KO}$=17<br><br>*Last three minutes are missing from one $MNK1^{KO}$ mouse*	Mixed-effects model	time: $F_{(5.368, 238.0)} = 2.066$, P=0.0655<br>genotype: $F_{(2, 45)} = 0.08049$, P=0.9228<br>time × genotype interaction: $F_{(18, 399)} = 1.324$, P=0.1684
3B	Mean $\log_2$ value relative to wild-type from 4 mice per genotype. Number of pairs: 9114 proteins / genotype	Pearson r	$MNK1^{KO}$ logFC vs $MNK2^{KO}$ logFC: r = 0.5574 (95% confidence interval 0.5430 – 0.5713), P<0.0001<br><br>$MNK1^{KO}$ logFC vs $MNK1/2^{DKO}$ logFC: r = 0.5186 (95% confidence interval 0.5034 – 0.5334), P<0.0001<br><br>$MNK2^{KO}$ logFC vs $MNK1/2^{DKO}$ logFC: r = 0.4770 (95% confidence interval 0.4610 – 0.4927), P<0.0001
4A	Mean $\log_2$ value relative to wild-type from 4 mice per genotype. Number of pairs: 9114 proteins / genotype	Pearson r	$MNK1^{KO}$ logFC vs $MNK2^{KO}$ logFC: r = 0.05323 (95% confidence interval 0.03273 – 0.07368), P<0.0001<br><br>$MNK1^{KO}$ logFC vs $MNK1/2^{DKO}$ logFC: r = 0.2309 (95% confidence interval 0. 2114 – 0.2502), P<0.0001<br><br>$MNK2^{KO}$ logFC vs $MNK1/2^{DKO}$ logFC: r = 0.6062 (95%

			confidence interval 0.5930 – 0.6190), P<0.0001
4E	Mean log_2 value relative to wild-type from 4 mice per genotype.<br>Ribosomes: 73<br>All: 73 randomly selected proteins	Kolmogorov-Smirnov test	$MNK1^{KO}$ CX: D= 0.3699, P<0.0001<br><br>$MNK2^{KO}$ CX: D= 0.3288, P=0.0007<br><br>$MNK1/2^{DKO}$ CX: D= 0.4110, P<0.0001<br><br>$MNK1^{KO}$ SYP: D= 0.6849, P<0.0001<br><br>$MNK2^{KO}$ SYP: D= 0.3288, P=0.0007<br><br>$MNK1/2^{DKO}$ SYP: D= 0.6164, P<0.0001
6B	wild-type=18<br>$MNK1^{KO}$=12<br>$MNK2^{KO}$=12	Kruskal-Wallis test, Dunn's post-hoc test for multiple comparisons	$K_{(3)}$=10.77, P=0.0046<br><br>wild-type vs $MNK1^{KO}$: P= 0.0142<br>wild-type vs $MNK2^{KO}$: P= 0.0234<br>$MNK1^{KO}$ vs $MNK2^{KO}$: P>0.9999
6D	wild-type=21<br>$MNK1^{KO}$=15<br>$MNK2^{KO}$=16	Kruskal-Wallis test	$K_{(3)}$=2.754, P=0.2523
6F	wild-type=5<br>$MNK1^{KO}$=5<br>$MNK2^{KO}$=5<br>$MNK1/2^{DKO}$=4	Two-way ANOVA	Subcellular region: $F_{(1, 30)}$ = 210.9, P<0.0001<br>genotype: $F_{(3, 30)}$ = 0.4374, P=0.7279<br>Subcellular region × genotype: $F_{(3, 30)}$ = 0.3395, P=0.7969
6H	wild-type=165 synapses (2 mice)<br>$MNK1^{KO}$=178 synapses (3 mice)<br>$MNK2^{KO}$=157 synapses (3 mice)	Kruskal-Wallis test, Dunn's post-hoc test for multiple comparisons	$K_{(3)}$= 8.594, P=0.0136<br><br>wild-type vs $MNK1^{KO}$: P= 0.0699<br>wild-type vs $MNK2^{KO}$: P= 0.0181<br>$MNK1^{KO}$ vs $MNK2^{KO}$: P>0.9999
6I	wild-type=165 synapses (2 mice)<br>$MNK1^{KO}$=178 synapses (3 mice)	Kruskal-Wallis test, Dunn's post-hoc test for multiple comparisons	$K_{(3)}$= 46.83, P<0.0001<br><br>wild-type vs $MNK1^{KO}$: P= 0.0202<br>wild-type vs $MNK2^{KO}$: P<0.0001<br>$MNK1^{KO}$ vs $MNK2^{KO}$: P<0.0001

	$MNK2^{KO}$=157<br>synapses (3<br>mice)		
6K	wild-type=14<br>$MNK1^{KO}$=14<br>$MNK2^{KO}$=16	One-way ANOVA, Tukey's post-hoc test for multiple comparisons	$F_{(2, 41)}$ = 17.27, P<0.0001<br><br>wild-type vs $MNK1^{KO}$: P= 0.0026<br>wild-type vs $MNK2^{KO}$: P< 0.0001<br>$MNK1^{KO}$ vs $MNK2^{KO}$: P= 0.0932
6L	wild-type=14<br>$MNK1^{KO}$=15<br>$MNK2^{KO}$=15	Kruskal-Wallis test, Dunn's post-hoc test for multiple comparisons	$K_{(3)}$=22.14, P<0.0001<br><br>wild-type vs $MNK1^{KO}$: P<0.0001<br>wild-type vs $MNK2^{KO}$: P=0.0166<br>$MNK1^{KO}$ vs $MNK2^{KO}$: P= 0.1545
6M	Mean $\log_2$ value relative to wild-type from 4 mice per genotype. Number of pairs: 10230 phosphosites / genotype	Pearson r	*cortex:*<br>$MNK1^{KO}$ logFC vs $MNK2^{KO}$ logFC: r = 0.4523 (95% confidence interval 0.4368 – 0.4676), P<0.0001<br><br>*synaptoneurosomes:*<br>$MNK1^{KO}$ logFC vs $MNK2^{KO}$ logFC: r = 0.2254 (95% confidence interval 0.2069 – 0.2437), P<0.0001
**Supplementary Figures**	**Sample Size**	**Statistical test**	**Values**
S1B	wild-type=17<br>$MNK1^{KO}$=14<br>$MNK2^{KO}$=17	RM two-way ANOVA	trial: $F_{(4, 180}$ = 85.86, P<0.0001<br>genotype: $F_{(2, 45)}$ = 0.7987, P=0.4562<br>trial × genotype: $F_{(8, 180)}$ = 1.933, P=0.0577
S1C	wild-type=17<br>$MNK1^{KO}$=14<br>$MNK2^{KO}$=17	One-way ANOVA, Tukey's post-hoc test for multiple comparisons	$F_{(2, 45)}$ = 6.565, P=0.0031<br>wild-type vs $MNK1^{KO}$: P=0.0038<br>wild-type vs $MNK2^{KO}$: P= 0.8385<br>$MNK1^{KO}$ vs $MNK2^{KO}$: P= 0.0164
S1D	wild-type=17<br>$MNK1^{KO}$=14<br>$MNK2^{KO}$=17<br><br>*Trial 5 is missing from one $MNK2^{KO}$ mouse*	*Nose to tail*<br>Mixed-effects model<br><br>*Nose to body*<br>Mixed-effects model, Tukey's post-hoc test for multiple comparisons	*Nose to tail*<br>trial: $F_{(2.676, 119.8)}$ = 30.94, P<0.0001<br>genotype: $F_{(2, 45)}$ = 2.411, P=0.1012<br>trial × genotype: $F_{(8, 179)}$ = 0.1849, P=0.9927<br><br>*Nose to body*<br>trial: $F_{(3.757, 168.1)}$ = 4.985, P=0.0010

			genotype: $F_{(2, 45)}$ = 4.599, P=0.0152<br>trial × genotype: $F_{(8, 179)}$ = 0.8569,<br>P=0.5541<br><br>wild-type vs MNK1^{KO}<br>Trial1: P= 0.0520<br>Trial2: P= 0.4134<br>Trial3: P= 0.9037<br>Trial4: P= 0.6319<br>Trial5: P= 0.3153<br><br>wild-type vs MNK2^{KO}<br>Trial1: P= 0.0155<br>Trial2: P= 0.0752<br>Trial3: P= 0.4734<br>Trial4: P= 0.1237<br>Trial5: P= 0.0995<br><br>MNK1^{KO} vs MNK2^{KO}<br>Trial1: P= 0.9999<br>Trial2: P= 0.6942<br>Trial3: P= 0.3563<br>Trial4: P= 0.5535<br>Trial5: P= 0.9831
		Nose to nose<br>Mixed-effects model	*Nose to nose*<br>trial: $F_{(2.235, 100.0)}$ = 5.231, P=0.0052<br>genotype: $F_{(2, 45)}$ = 1.827, P=0.1726<br>trial × genotype: $F_{(8, 179)}$ = 1.186, P=0.3099
S1E	wild-type=17<br>MNK1^{KO}=14<br>MNK2^{KO}=17<br><br>*Trial 5 is missing from one MNK2^{KO} mouse*	*Side by side*<br>Mixed-effects model, Tukey's post-hoc test for multiple comparisons	*Side by side*<br>trial: $F_{(2.492, 111.5)}$ = 3.214, P=0.0336<br>genotype: $F_{(2, 45)}$ = 3.387, P=0.0426<br>trial × genotype: $F_{(8, 179)}$ = 0.7083, P=0.6840<br><br>wild-type vs MNK1^{KO}<br>Trial1: P= 0.0714<br>Trial2: P= 0.0610<br>Trial3: P= 0.9046<br>Trial4: P= 0.7391<br>Trial5: P= 0.1718<br><br>wild-type vs MNK2^{KO}<br>Trial1: P= 0.2334<br>Trial2: P= 0.4526

			Trial3: P= 0.5697<br>Trial4: P= 0.3383<br>Trial5: P= 0.9498<br><br>MNK1^{KO} vs MNK2^{KO}<br>Trial1: P= 0.7469<br>Trial2: P= 0.6209<br>Trial3: P= 0.7352<br>Trial4: P= 0.7417<br>Trial5: P= 0.2276
		Side by side reverse<br>Mixed-effects model, Tukey's post-hoc test for multiple comparisons	*Side by side reverse*<br>trial: $F_{(3.672, 164.3)} = 1.060$, P=0.3753<br>genotype: $F_{(2, 45)} = 5.983$, P=0.0050<br>trial $\times$ genotype: $F_{(8, 179)} = 0.5915$, P=0.7841<br><br>wild-type vs MNK1^{KO}<br>Trial1: P= 0.2486<br>Trial2: P= 0.0274<br>Trial3: P= 0.3846<br>Trial4: P= 0.9537<br>Trial5: P= 0.1072<br><br>wild-type vs MNK2^{KO}<br>Trial1: P= 0.3977<br>Trial2: P= 0.1633<br>Trial3: P= 0.2106<br>Trial4: P= 0.4885<br>Trial5: P= 0.4039<br><br>MNK1^{KO} vs MNK2^{KO}<br>Trial1: P= 0.8071<br>Trial2: P= 0.3965<br>Trial3: P= 0.9997<br>Trial4: P= 0.7163<br>Trial5: P= 0.8183
S1F	wild-type=17<br>MNK1^{KO}=14<br>MNK2^{KO}=17<br><br>*Trial 5 is missing from one MNK2^{KO} mouse*	*Sniffing test*<br>Mixed-effects model, Tukey's post-hoc test for multiple comparisons	*Sniffing test*<br>trial: $F_{(2.962, 132.5)} = 3.233$, P=0.0250<br>genotype: $F_{(2, 45)} = 12.11$, P<0.0001<br>trial $\times$ genotype: $F_{(8, 179)} = 1.633$, P= 0.1182<br><br>wild-type vs MNK1^{KO}<br>Trial1: P= 0.5450<br>Trial2: P= 0.0354<br>Trial3: P= 0.3064<br>Trial4: P= 0.0154<br>Trial5: P= 0.2479

			wild-type vs MNK2[KO]<br>Trial1: P= 0.0001<br>Trial2: P= 0.0008<br>Trial3: P= 0.0182<br>Trial4: P= 0.2998<br>Trial5: P= 0.0304<br><br>MNK1[KO] vs MNK2[KO]<br>Trial1: P= 0.0472<br>Trial2: P= 0.3948<br>Trial3: P= 0.3763<br>Trial4: P= 0.1547<br>Trial5: P= 0.8933
		Climbing test<br>Mixed-effects model, Tukey's post-hoc test for multiple comparisons	*Climbing test*<br>trial: $F_{(3.078, 137.8)} = 1.141$, P=0.3355<br>genotype: $F_{(2, 45)} = 5.391$, P=0.0080<br>trial $\times$ genotype: $F_{(8, 179)} = 0.5523$, P= 0.8157<br><br>wild-type vs MNK1[KO]<br>Trial1: P= 0.3453<br>Trial2: P= 0.1734<br>Trial3: P= 0.4833<br>Trial4: P= 0.2026<br>Trial5: P= 0.0955<br><br>wild-type vs MNK2[KO]<br>Trial1: P= 0.0298<br>Trial2: P= 0.0693<br>Trial3: P= 0.2653<br>Trial4: P= 0.6798<br>Trial5: P= 0.7745<br><br>MNK1[KO] vs MNK2[KO]<br>Trial1: P= 0.9522<br>Trial2: P= 0.9916<br>Trial3: P= 0.8581<br>Trial4: P= 0.5523<br>Trial5: P= 0.3675
		Speed test<br>Mixed-effects model, Tukey's post-hoc test for multiple comparisons	*Speed test*<br>trial: $F_{(3.629, 162.4)} = 17.83$, P<0.0001<br>genotype: $F_{(2, 45)} = 1.410$, P=0.2548<br>trial $\times$ genotype: $F_{(8, 179)} = 0.9646$, P= 0.4652

S1G	*Trial 1-4*<br>wild-type=17<br>MNK1^{KO}=14<br>MNK2^{KO}=17<br><br>*Trial 5*<br>wild-type=17<br>MNK1^{KO}=14<br>MNK2^{KO}=16<br><br>*Trial 5 is missing from one MNK2^{KO} mouse*	*Nose to tail stimulus*<br>*Trial 1-4*<br>RM two-way ANOVA<br>Tukey's post-hoc test for multiple comparisons<br><br>*Trial 5*<br>Kruskal-Wallis test, Dunn's post-hoc test for multiple comparisons<br><br>*Nose to body stimulus*<br>*Trial 1-4*<br>RM two-way ANOVA<br>Tukey's post-hoc test for multiple comparisons<br><br>*Trial 5*<br>Kruskal-Wallis test, Dunn's post-hoc test for multiple comparisons	*Nose to tail stimulus*<br>trial: $F_{(2.405, 108.2)}$ = 9.880, P<0.0001<br>genotype: $F_{(2, 45)}$ = 0.1016, P=0.9036<br>trial × genotype: $F_{(6, 135)}$ = 0.2036, P=0.9752<br><br>*Trial 5*<br>$K_{(3)}$= 1.752, P= 0.4164<br><br>*Nose to body stimulus*<br>trial: $F_{(1.831, 82.39)}$ = 1.237, P=0.2933<br>genotype: $F_{(2, 45)}$ = 1.049, P=0.3587<br>trial × genotype: $F_{(6, 135)}$ = 0.7871, P=0.5815<br><br>*Trial 5*<br>$K_{(3)}$= 1.133, P= 0.5676
S1H	*Trial 1-4*<br>wild-type=17<br>MNK1^{KO}=14<br>MNK2^{KO}=17<br>*Trial 5*<br>wild-type=17<br>MNK1^{KO}=14<br>MNK2^{KO}=16<br><br>*Trial 5 is missing from one MNK2^{KO} mouse*	*Sniffing stimulus*<br>*Trial 1-4*<br>RM two-way ANOVA<br>Tukey's post-hoc test for multiple comparisons<br><br>*Trial 5*<br>Kruskal-Wallis test, Dunn's post-hoc test for multiple comparisons	*Sniffing stimulus*<br>trial: $F_{(2.726, 122.7)}$ = 0.8485, P=0.4608<br>genotype: $F_{(2, 45)}$ = 4.349, P=0.0188<br>trial × genotype: $F_{(6, 135)}$ = 1.347, P=0.2405<br><br>wild-type vs MNK1^{KO}<br>Trial1: P= 0.2406<br>Trial2: P= 0.0848<br>Trial3: P= 0.8704<br>Trial4: P= 0.5397<br><br>wild-type vs MNK2^{KO}<br>Trial1: P= 0.0840<br>Trial2: P= 0.0009<br>Trial3: P= 0.1845<br>Trial4: P= 0.7983<br><br>MNK1^{KO} vs MNK2^{KO}<br>Trial1: P= 0.8235<br>Trial2: P= 0.5235<br>Trial3: P= 0.4831<br>Trial4: P= 0.8956<br><br>*Trial 5*<br>$K_{(3)}$= 7.106, P= 0.0286<br>wild-type vs MNK1^{KO}: P= 0.1021

			wild-type vs MNK2^{KO}: P= 0.0465<br>MNK1^{KO} vs MNK2^{KO}: P >0.9999
		Climbing stimulus<br>*Trial 1-4*<br>RM two-way ANOVA<br>Tukey's post-hoc test for multiple comparisons<br><br>*Trial 5*<br>Kruskal-Wallis test, Dunn's post-hoc test for multiple comparisons	*Climbing stimulus*<br>trial: $F_{(2.199, 98.94)}$ = 13.31, P<0.0001<br>genotype: $F_{(2, 45)}$ = 9.062, P=0.0005<br>trial $\times$ genotype: $F_{(6, 135)}$ = 1.747, P=0.1149<br><br>wild-type vs MNK1^{KO}<br>Trial1: P= 0.0098<br>Trial2: P= 0.0122<br>Trial3: P= 0.0029<br>Trial4: P= 0.3759<br><br>wild-type vs MNK2^{KO}<br>Trial1: P= 0.1692<br>Trial2: P= 0.1393<br>Trial3: P= 0.0425<br>Trial4: P>0.9999<br><br>MNK1^{KO} vs MNK2^{KO}<br>Trial1: P= 0.6840<br>Trial2: P= 0.2173<br>Trial3: P= 0.5598<br>Trial4: P= 0.2081<br><br>*Trial 5*<br>$K_{(3)}$= 8.680, P= 0.0130<br>wild-type vs MNK1^{KO}: P= 0.0100<br>wild-type vs MNK2^{KO}: P= 0.7436<br>MNK1^{KO} vs MNK2^{KO}: P = 0.2177
		Speed stimulus<br>*Trial 1-4*<br>RM two-way ANOVA<br>Tukey's post-hoc test for multiple comparisons<br>*Trial 5*<br>Kruskal-Wallis test, Dunn's post-hoc test for multiple comparisons	*Speed stimulus*<br>trial: $F_{(2.267, 102.0)}$ = 48.06, P<0.0001<br>genotype: $F_{(2, 45)}$ = 3.162, P=0.0519<br>trial $\times$ genotype: $F_{(6, 135)}$ = 3.638, P=0.0022<br><br>wild-type vs MNK1^{KO}<br>Trial1: P= 0.0014<br>Trial2: P= 0.0247<br>Trial3: P= 0.0725<br>Trial4: P= 0.7854<br><br>wild-type vs MNK2^{KO}<br>Trial1: P= 0.9622<br>Trial2: P= 0.9987<br>Trial3: P= 0.6026

			Trial4: P= 0.8590<br><br>MNK1^{KO} vs MNK2^{KO}<br>Trial1: P= 0.0272<br>Trial2: P= 0.0407<br>Trial3: P= 0.4160<br>Trial4: P= 0.9713<br><br>*Trial 5*<br>$K_{(3)}$= 6.324, P= 0.0423<br>wild-type vs MNK1^{KO}: P= 0.1265<br>wild-type vs MNK2^{KO}: P>0.9999<br>MNK1^{KO} vs MNK2^{KO}: P = 0.0570
S1I	wild-type=17<br>MNK1^{KO}=15<br>MNK2^{KO}=17	RM two-way ANOVA	trial: $F_{(3.039, 136.8)}$ = 67.06, P<0.0001<br>genotype: $F_{(2, 45)}$ = 0.8909, P=0.4174<br>trial × genotype: $F_{(8, 180)}$ = 1.699, P=0.1013
S1J	wild-type=17<br>MNK1^{KO}=15<br>MNK2^{KO}=17	One-way ANOVA, Tukey's post-hoc test for multiple comparisons	$F_{(2, 45)}$ = 6.475, P=0.0034<br>wild-type vs MNK1^{KO}: P= 0.0423<br>wild-type vs MNK2^{KO}: P= 0.5220<br>MNK1^{KO} vs MNK2^{KO}: P = 0.0027
S2A-B	*females:*<br>wild-type=10<br>MNK1^{KO}=8<br>MNK2^{KO}=12<br><br>*males:*<br>wild-type=7<br>MNK1^{KO}=6<br>MNK2^{KO}=5	Three-way Repeated Measures ANOVA	Genotype: $F_{(2, 42)}$=0.28, P=0.760<br>Sex: $F_{(1, 42)}$=1.19, P=0.2820<br>genotype x Sex $F_{(2, 42)}$=1.63, P=0.2080<br>trials: $F_{(3.35, 140.79)}$=81.16, P<0.0001<br>genotype x trials: $F_{(6.7, 140.79)}$=1.887, P=0.0820<br>Sex x trials $_{(3.35, 140.79)}$: F=0.86, P=0.4720<br>Sex x trials x genotype $_{(6.7, 140.79)}$: F=0.67, P=0.6880
S2C	*females:*<br>wild-type=10<br>MNK1^{KO}=8<br>MNK2^{KO}=12<br><br>*males:*<br>wild-type=7<br>MNK1^{KO}=6<br>MNK2^{KO}=5	Two-way ANOVA, Tukey's post-hoc test for multiple comparisons	Interaction: $F_{(2, 42)}$ = 0.2222, P=0.8017<br>Sex: $F_{(1, 42)}$ = 0.2822, P=0.5980<br>Genotype: $F_{(2, 42)}$ = 6.141, P=0.0046<br><br>wild-type vs MNK1^{KO}: P= 0.0060<br>wild-type vs MNK2^{KO}: P= 0.9260<br>MNK1^{KO} vs MNK2^{KO}: P = 0.0209
S2D-E	*females:*<br>wild-type=10<br>MNK1^{KO}=8	Three-way Repeated Measures ANOVA	Genotype: $F_{(2, 43)}$ =2.48, P=0.0950<br>Sex: $F_{(1, 43)}$ =2.23, P=0.1430

	$MNK2^{KO}$=12<br><br>*males:*<br>wild-type=7<br>$MNK1^{KO}$=7<br>$MNK2^{KO}$=5		genotype x sex: $F_{(2, 42)}$ =2.06, P=0.139<br>trials: $F_{(3.87, 166.45)}$ =42.84, P<0.0001<br>genotype x trials: $F_{(7.74, 166.45)}$ =1.47, P=0.175<br>Sex x trials: $F_{(3.87, 166.45)}$ =0.58, P=0.669<br>Sex x trials x genotype: $F_{(7.74, 166.45)}$ =1.62, P=0.125
S2F-G	*females:*<br>wild-type=10<br>$MNK1^{KO}$=8<br>$MNK2^{KO}$=12<br><br>*males:*<br>wild-type=7<br>$MNK1^{KO}$=7<br>$MNK2^{KO}$=5	Three-way Repeated Measures ANOVA	Genotype: $F_{(2, 43)}$ =9.80, P<0.0001<br>Sex: $F_{(1, 43)}$ =0.54, P=0.465<br>genotype x sex: $F_{(2, 43)}$ =1.74, P=0.188<br>trials: $F_{(2.43, 104.6)}$ =34.96, P<0.0001<br>genotype x trials: $F_{(4.86, 104.6)}$ =5.89, P<0.0001<br>Sex x trials: $F_{(2.43, 104.6)}$ =0.83, P=0.461<br>Sex x trials x genotype: $F_{(4.86, 104.6)}$ =0.61, P=0.689
S2H	*females:*<br>wild-type=10<br>$MNK1^{KO}$=8<br>$MNK2^{KO}$=12<br><br>*males:*<br>wild-type=7<br>$MNK1^{KO}$=7<br>$MNK2^{KO}$=5	Two-way ANOVA, Tukey's post-hoc test for multiple comparisons	Interaction: $F_{(2, 43)}$ = 0.9540, P=0.3932<br>Sex: $F_{(1, 43)}$ = 0.6882, P=0.4114<br>Genotype: $F_{(2, 43)}$ = 14.51, P<0.0001<br><br>Wild-type vs $MNK1^{KO}$: P=0.0221<br>Wild-type vs $MNK2^{KO}$: P=0.0211<br>$MNK1^{KO}$ vs $MNK2^{KO}$: P<0.0001
S2I	*females:*<br>wild-type=10<br>$MNK1^{KO}$=8<br>$MNK2^{KO}$=12<br><br>*males:*<br>wild-type=7<br>$MNK1^{KO}$=7<br>$MNK2^{KO}$=5	Three-way Repeated Measures ANOVA, Bonferroni's post-hoc test for multiple comparisons	Genotype: $F_{(2, 43)}$ =11.66, P<0.0001<br>Sex: $F_{(1, 43)}$ =0.51, P=0.479<br>Novelty: $F_{(1, 43)}$ =21.05, P<0.0001<br>genotype x sex: $F_{(2, 43)}$ =3.23, P=0.049<br>genotype x novelty: $F_{(2, 43)}$ =4.62, P=0.015<br>Sex x novelty: $F_{(1, 43)}$ =0.70, P=0.409<br>Sex x novelty x genotype: $F_{(2, 43)}$ =0.52, P=0.596<br><br>Post-hoc: Novelty x genotype<br>*Familiar vs novel object*<br>Wild-type: P=0.004<br>$MNK1^{KO}$: P=0.772

			MNK2^{KO}: P=0.003<br><br><u>Post-hoc: Novelty x sex</u><br>*female*<br>Wild-type vs MNK1^{KO} : P=0.0056<br>Wild-type vs MNK2^{KO}: P=0.056<br>MNK1^{KO} vs MNK2^{KO}: P<0.0001<br><br>*male*<br>Wild-type vs MNK1^{KO}: P=0.158<br>Wild-type vs MNK2^{KO}: P>0.9999<br>MNK1^{KO} vs MNK2^{KO}: P=0.687<br><br><u>Post-hoc: Novelty x sex</u><br>*Female vs male*<br>Wild-type: P=0.38<br>MNK1^{KO}: P=0.017<br>MNK2^{KO}: P=0.115
S2J	females:<br>wild-type=10<br>MNK1^{KO}=8<br>MNK2^{KO}=12<br><br>males:<br>wild-type=7<br>MNK1^{KO}=7<br>MNK2^{KO}=5	Two-way ANOVA, Tukey's post-hoc test for multiple comparisons	Interaction: $F_{(2, 43)}$ = 0.8828, P=0.4210<br>Sex: $F_{(1, 43)}$ = 1.423, P=0.2394<br>Genotype: $F_{(2, 43)}$ = 5.574, P=0.0070<br><br>Wild-type vs MNK1^{KO} : P=0.0497<br>Wild-type vs MNK2^{KO}: P=0.6531<br>MNK1^{KO} vs MNK2^{KO}: P=0.0071
S2K	females:<br>wild-type=10<br>MNK1KO=6<br>MNK2KO=12<br><br>males:<br>wild-type=6<br>MNK1^{KO}=7<br>MNK2^{KO}=5	Two-way ANOVA	Interaction: $F_{(2, 40)}$ = 2.049, P=0.1422<br>Sex: $F_{(1, 40)}$ = 0.01534, P=0.9020<br>Genotype: $F_{(2, 40)}$ = 2.453, P=0.0989
S2L	females:<br>wild-type=10<br>MNK1^{KO}=6<br>MNK2^{KO}=12<br><br>males:<br>wild-type=6<br>MNK1^{KO}=7<br>MNK2^{KO}=5	Two-way ANOVA	Interaction: $F_{(2, 40)}$ = 0.1024, P=0.9029<br>Sex: $F_{(1, 40)}$ = 2.415, P=0.1281<br>Genotype: $F_{(2, 40)}$ = 0.1471, P=0.8637
S3A	wild-type= 13<br>MNK1/2^{DKO}=15	Two-way Repeated Measures ANOVA	Genotype x trial: $F_{(4, 104)}$ = 0.2954, P=0.8804<br>Trial: $F_{(2.730, 70.97)}$ = 27.66, P<0.0001

			Genotype: $F_{(1, 26)}$ = 0.2456, P=0.6243
S3B	wild-type= 13<br>MNK1/2^{DKO}=15	Unpaired t-test	t=0.1726, df=26<br>P=0.8635
S3C	wild-type= 13<br>MNK1/2^{DKO}=16	Two-way Repeated Measures ANOVA	Genotype x trial: $F_{(11, 297)}$ = 0.3062, P=0.9843<br>Trial: $F_{(3.989, 107.7)}$ = 19.90, P<0.0001<br>Genotype: $F_{(1, 27)}$ = 0.6022, P=0.4445
S3D	wild-type= 13<br>MNK1/2^{DKO}=16	Two-way Repeated Measures ANOVA	Genotype x trial: $F_{(4, 108)}$ = 0.9991, P=0.4114<br>Trial: $F_{(2.182, 58.92)}$ = 21.69, P<0.0001<br>Genotype: $F_{(1, 27)}$ = 0.8417, P=0.3670
S3E	wild-type= 13<br>MNK1/2^{DKO}=16	Unpaired t-test	t=0.09051, df=27<br>P=0.9286
S3F	wild-type= 13<br>MNK1/2^{DKO}=16	Two-way Repeated Measures ANOVA, Šídák's post-hoc test for multiple comparisons	Genotype x trial: $F_{(1, 27)}$ = 0.2934, P=0.5925<br>Trial: $F_{(1, 27)}$ = 27.39, P<0.0001<br>Genotype: $F_{(1, 27)}$ = 0.1725, P=0.6812<br><br>Familiar vs novel object:<br>wild-type: P=0.0078<br>MNK1/2$^{DKO:}$ P=0.0004
S3G	wild-type= 13<br>MNK1/2^{DKO}=16	Mann-Whitney test	U=104<br>P>0.9999
S3H	wild-type= 13<br>MNK1/2^{DKO}=16	Two-way Repeated Measures ANOVA, Tukey's post-hoc test for multiple comparisons	Genotype x time: $F_{(9, 243)}$ = 1.131, P=0.3416<br>Time: $F_{(4.908, 132.5)}$ = 6.144, P<0.0001<br>Genotype: $F_{(1, 27)}$ = 18.34, P=0.0002<br><br>wild-type vs MNK1/2^{DKO}<br>Minute 1: P=0.0035<br>Minute 2: P=0.0104<br>Minute 3: P=0.0437<br>Minute 4: P=0.0335<br>Minute 5: P=0.0343<br>Minute 6: P=0.0404<br>Minute 7: P=0.1042<br>Minute 8: P=0.4245<br>Minute 9: P=0.3170<br>Minute 10: P=0.1799
S3I	wild-type= 13<br>MNK1/2^{DKO}=16	Two-way Repeated Measures ANOVA	Genotype x time: $F_{(9, 243)}$ = 0.4459, P=0.9089

			Time: $F_{(4.466, 120.6)}$ = 2.781, P=0.0250<br>Genotype: $F_{(1, 27)}$ = 0.2810, P=0.6004
S3J	wild-type= 13<br>MNK1/2^{DKO}=15	Two-way Repeated Measures ANOVA	*Nose to tail:*<br>Genotype x trial: $F_{(4, 104)}$ = 2.360, P=0.0581<br>Trial: $F_{(2.535, 65.91)}$ = 19.31, P<0.0001<br>Genotype: $F_{(1, 26)}$ = 0.8639, P=0.3612<br><br>*Nose to body:*<br>Genotype x trial: $F_{(4, 104)}$ = 0.7983, P=0.5290<br>Trial: $F_{(3.376, 87.78)}$ = 3.135, P=0.0247<br>Genotype: $F_{(1, 26)}$ = 0.9439, P=0.3402<br><br>*Nose to nose:*<br>Genotype x trial: $F_{(4, 104)}$ = 0.8784, P=0.4796<br>Trial: $F_{(2.175, 56.56)}$ = 0.6077, P=0.5615<br>Genotype: $F_{(1, 26)}$ = 0.01554, P=0.9018
S3K	wild-type= 13<br>MNK1/2^{DKO}=15	Two-way Repeated Measures ANOVA	*Side by side:*<br>Genotype x trial: $F_{(4, 104)}$ = 0.3384, P=0.8515<br>Trial: $F_{(2.790, 72.53)}$ = 3.958, P=0.0131<br>Genotype: $F_{(1, 26)}$ = 0.002354, P=0.9617<br><br>*Side by side reverse:*<br>Genotype x trial: $F_{(4, 104)}$ = 0.3721, P=0.8281<br>Trial: $F_{(3.338, 86.79)}$ = 4.022, P=0.0077<br>Genotype: $F_{(1, 26)}$ = 0.05315, P=0.8195
S3L	wild-type= 13<br>MNK1/2^{DKO}=15	Two-way Repeated Measures ANOVA, Tukey's post-hoc test for multiple comparisons	*Sniffing test:*<br>Genotype x trial: $F_{(4, 104)}$ = 0.7951, P=0.5310<br>Trial: F ($_{2.426, 63.09)}$ = 0.8449, P=0.4537<br>Genotype: $F_{(1, 26)}$ = 3.936, P=0.0579

			Climbing test:<br>Genotype x trial: $F_{(4, 104)}$ = 0.7891, P=0.5348<br>Trial: $F_{(2.636, 68.55)}$ = 0.3906, P=0.7345<br>Genotype: $F_{(1, 26)}$ = 5.317, P=0.0293<br><br>wild-type vs MNK1/2^{DKO}<br>Trial 1: P=0.8295<br>Trial 2: P=0.3322<br>Trial 3: P=0.2598<br>Trial 4: P=0.0716<br>Trial 5: P=0.0336<br><br>*Speed:*<br>Genotype x trial: $F_{(4, 104)}$ = 0.7477, P=0.5617<br>Trial: $F_{(3.310, 86.06)}$ = 7.521, P<0.0001<br>Genotype: $F_{(1, 26)}$ = 0.3190, P=0.5770
S3M	wild-type= 13<br>MNK1/2^{DKO}=15	*Trial 1-4*<br>RM two-way ANOVA<br>Tukey's post-hoc test for multiple comparisons<br>*Trial 5*<br>Mann Whitney test	*Nose to tail stimulus:*<br>*Trial 1-4*<br>Genotype x trial: $F_{(3, 78)}$ = 1.480, P=0.2264<br>Trial: $F_{(2.643, 68.71)}$ = 8.680, P=0.0001<br>Genotype: $F_{(1, 26)}$ = 0.4082, P=0.5285<br>*Trial 5*<br>U=58.50, P=0.0731<br><br>*Nose to body stimulus:*<br>*Trial 1-4*<br>Genotype x trial: $F_{(3, 78)}$ = 2.327, P=0.0811<br>Trial: $F_{(2.684, 69.78)}$ = 1.908, P=0.1424<br>Genotype: $F_{(1, 26)}$ = 0.6106, P=0.4416<br>*Trial 5*<br>U=96, P=0.9639
S3N	wild-type= 13<br>MNK1/2^{DKO}=15	*Sniffing stimulus:*<br>*Trial 1-4*<br>RM two-way ANOVA<br>Tukey's post-hoc test for multiple comparisons	*Sniffing stimulus:*<br>*Trial 1-4*<br>Genotype x trial: $F_{(3, 78)}$ = 0.4744, P=0.7010<br>Trial: $F_{(2.283, 59.36)}$ = 0.3091,

		Trial 5<br>Unpaired t-test<br><br><br><br><br>*Climbing stimulus:*<br>*Trial 1-4*<br>RM two-way ANOVA<br>Tukey's post-hoc test for multiple comparisons<br>*Trial 5*<br>Unpaired t-test<br><br><br><br><br>*Speed stimulus:*<br>*Trial 1-4*<br>RM two-way ANOVA<br>Tukey's post-hoc test for multiple comparisons<br>*Trial 5*<br>Unpaired t-test	P=0.7635<br>Genotype: $F_{(1, 26)}$ = 1.249, P=0.2739<br><br>*Trial 5*<br>t=0.2977, df=26, P=0.7683<br><br>*Climbing stimulus:*<br>*Trial 1-4*<br>Genotype x trial: F (3, 78) = 0.2593, P=0.8545<br>Trial: $F_{(2.485, 64.62)}$ = 3.488, P=0.0275<br>Genotype: $F_{(1, 26)}$ = 1.224, P=0.2786<br><br>*Trial 5*<br>t=1.495, df=26, P=0.1469<br><br>*Speed stimulus:*<br>*Trial 1-4*<br>Genotype x trial: $F_{(3, 78)}$ = 0.06861, P=0.9765<br>Trial: $F_{(2.731, 71.02)}$ = 15.05, P<0.0001<br>Genotype: $F_{(1, 26)}$ = 3.580, P=0.0697<br><br>*Trial 5*<br>U=69, P=0.2005
S3O	wild-type= 13<br>MNK1/2^{DKO}=16	Mixed-effects model, Tukey's post-hoc test for multiple comparisons	Genotype x trial: $F_{(4, 107)}$ = 0.6498, P= 0.6282<br>Trial: $F_{(2.370, 63.41)}$ = 4.601, P=0.0097<br>Genotype: $F_{(1, 27)}$ = 14.24, P= 0.0008<br><br>wild-type vs MNK1/2^{DKO}<br>Trial 1: P=0.0032<br>Trial 2: P=0.1093<br>Trial 3: P<0.0001<br>Trial 4: P=0.0943<br>Trial 5: P=0.1019
S3P	wild-type= 13<br>MNK1/2^{DKO}=16	Mann Whitney test	U=79, P=0.2875
S5B	Mean $\log_2$ value of ETC-168 treated wild-type relative to vehicle-treated wild-type from 4	Kolmogorov-Smirnov test	WT ETC-168: D= 0.3506, P=0.0002

	mice per treatment.<br>Ribosomes: 77<br>All: 77 randomly selected proteins		
S5B	Ribosomal proteins: 30<br>All: 680	Wilcoxon-Mann–Whitney U test	W = 6762, P = 0.001379
S6C	Mean log_2 value relative to wild-type from 3 mice per genotype.<br>Number of pairs: 18141 RNAs / genotype	Pearson r	$MNK1^{KO}$ logFC vs $MNK2^{KO}$ logFC: r = 0.2649 (95% confidence interval 0.2513 – 0.2784), P<0.0001
S6D	logFC value of $MNK1^{KO}$ relative to wild-type from 3 (mRNA) or 4 (protein) mice per genotype.<br>Number of pairs: 8294 genes/proteins	Pearson r	$MNK1^{KO}$ logFC protein vs $MNK1^{KO}$ logFC RNA: r = -0.02401 (95% confidence interval: -0.04551 to -0.002487), P=0.0288
S6E	logFC value of $MNK2^{KO}$ relative to wild-type from 3 (mRNA) or 4 (protein) mice per genotype.<br>Number of pairs: 8294 genes/proteins	Pearson r	$MNK2^{KO}$ logFC protein vs $MNK2^{KO}$ logFC RNA: r = 0.09438 (95% confidence interval: 0.07301 to 0.1157), P<0.0001
S6H	wild-type= 18<br>$MNK1^{KO}$=18<br>$MNK2^{KO}$=18<br>$MNK1/2^{DKO}$=18<br><br>N= mean fluorescent intensity of neurons per image. 5-7 images were analysed per animal, with 3 animals per genotype.	Kruskal-Wallis test	$K_{(4)}$= 3.024, P=0.3880
S6I	wild-type= 8<br>$MNK1^{KO}$=7<br>$MNK2^{KO}$=11<br>$MNK1/2^{DKO}$=5	Kruskal-Wallis test	$K_{(4)}$= 1.930, P=0.5870

S6J	wild-type= 10<br>MNK1^{KO}=7<br>MNK2^{KO}=8<br>MNK1/2^{DKO}=7	Kruskal-Wallis test	$K_{(4)}$= 2.694, P=0.4412
S7C	wild-type=6<br>MNK1^{KO}=7<br>MNK2^{KO}=7	Kruskal-Wallis test, Dunn's post-hoc test for multiple comparisons	$K_{(3)}$=12.46, P=0.0002<br>wild-type vs MNK1^{KO}: P=0.0200<br>wild-type vs MNK2^{KO}: P=0.0023<br>MNK1^{KO} vs MNK2^{KO}: P>0.9999
S7D	wild-type=6<br>MNK1^{KO}=7<br>MNK2^{KO}=7	Kruskal-Wallis test, Dunn's post-hoc test for multiple comparisons	$K_{(3)}$=15.43, P<0.0001<br>wild-type vs MNK1^{KO}: P=0.0003<br>wild-type vs MNK2^{KO}: P=0.0950<br>MNK1^{KO} vs MNK2^{KO}: P=0.1920
S7E	wild-type=14<br>MNK1^{KO}=14<br>MNK2^{KO}=16<br>MNK1/2^{DKO}=8	One-way ANOVA	F $_{F\ (3,\ 48)}$ = 2.378, P=0.0814
S7F	wild-type=14<br>MNK1^{KO}=15<br>MNK2^{KO}=15<br>MNK1/2^{DKO}=8	One-way ANOVA	$F_{(3,\ 48)}$ = 1.523, P=0.2204
S7G	wild-type=8<br>MNK1^{KO}=6<br>MNK2^{KO}=9<br>MNK1/2^{DKO}=9	One-way ANOVA, Tukey's post-hoc test for multiple comparisons	$F_{(3,\ 28)}$ = 4.855, P=0.0076<br><br>wild-type vs MNK1^{KO}: P= 0.0038<br>wild-type vs MNK2^{KO}: P= 0.3164<br>wild-type vs MNK1/2^{DKO}: P= 0.1924<br>MNK1^{KO} vs MNK2^{KO}: P= 0.1274<br>MNK1^{KO} vs MNK1/2^{DKO}: P= 0.2121<br>MNK2^{KO} vs MNK1/2^{DKO}: P= 0.9893
S7H	wild-type=9<br>MNK1^{KO}=9<br>MNK2^{KO}=11<br>MNK1/2^{DKO}=7	One-way ANOVA, Tukey's post-hoc test for multiple comparisons	$F_{(3,\ 32)}$ = 7.421, P=0.0007<br><br>wild-type vs MNK1^{KO}: P= 0.0054<br>wild-type vs MNK2^{KO}: P= 0.0022<br>wild-type vs MNK1/2^{DKO}: P= 0.8317<br>MNK1^{KO} vs MNK2^{KO}: P= 0.9985<br>MNK1^{KO} vs MNK1/2^{DKO}: P= 0.0738<br>MNK2^{KO} vs MNK1/2^{DKO}: P= 0.0417
S7I	wild-type=8<br>MNK1^{KO}=8<br>MNK2^{KO}=9	One-way ANOVA	$F_{(2,\ 22)}$ = 0.9563, P=0.3997
S7J	wild-type=9<br>MNK1^{KO}=9	One-way ANOVA	$F_{(3,\ 31)}$ = 0.7150, P=0.5505

	$MNK2^{KO}$=10<br>$MNK1/2^{DKO}$=7		
S7K	wild-type=6<br>$MNK1^{KO}$=7<br>$MNK2^{KO}$=7<br>$MNK1/2^{DKO}$=4	One-way ANOVA	$F_{(3, 20)}$ = 1.050, P=0.3922
S7L	wild-type=14<br>$MNK2^{KO}$=16	Mann-Whitney test	Mann-Whitney U = 52, P=0.0118
S7M	wild-type=8<br>$MNK2^{KO}$=9	Mann-Whitney test	Mann-Whitney U = 19, P=0.1139
